# Supplementary material for: Structure, mechanism, and evolution of the last step in vitamin C biosynthesis
Source: Nat Commun. 2024 May 16;15:4158. doi: 10.1038/s41467-024-48410-1 (PMC11099136; doi:10.1038/s41467-024-48410-1)
Supplement: Supplementary file 1 — Supplementary Information [file 41467_2024_48410_MOESM1_ESM.pdf]

# Supplementary Information

## Structure, mechanism, and evolution of the last step in vitamin C biosynthesis

Alessandro Boverio[1,2], Neelam Jamil[2], Barbara Mannucci[3], Maria Laura Mascotti[1,4,5*], Marco W. Fraaije[1*], Andrea Mattevi[2*]

[1] Molecular Enzymology group, University of Groningen, Nijenborgh 4, 9747AG Groningen, The Netherlands

[2] Department of Biology and Biotechnology, University of Pavia, via Ferrata 9, 27100 Pavia, Italy

[3] Centro Grandi Strumenti, University of Pavia, Via Bassi 21, 27100, Pavia, Italy.

[4] IMIBIO-SL CONICET, Facultad de Química Bioquímica y Farmacia, Universidad Nacional de San Luis, San Luis, Argentina.

[5] Present address: Instituto de Histología y Embriología de Mendoza (IHEM)-CONICET-Universidad Nacional de Cuyo, 5500 Mendoza, Argentina

*Correspondence to: mlmascotti@mendoza-conicet.gob.ar, m.w.fraaije@rug.nl, andrea.mattevi@unipv.it

## Table of contents

**Supplementary Figures** **Pages 3-23**

**SF1.** Fully annotated phylogeny of the eukaryotic aldonolactone oxidoreductases
**SF2.** Amino acid sequences of the reconstructed aldonolactone oxidoreductases
**SF3.** Absorption spectra of reconstructed enzymes
**SF4.** SDS-PAGE gels of GalDHs and mGULO
**SF5.** Effect of ionic strength and pH on vGalDH
**SF6.** Steady-state kinetics for vGalDH
**SF7.** Steady-state kinetics for vGalDH A113G oxidase activity
**SF8.** Steady-state kinetics for vGalDH A113G dehydrogenase activity
**SF9.** Steady-state kinetics for vGalDH G413N
**SF10.** Steady-state kinetics for cGULO
**SF11.** Steady-state kinetics for mGULO
**SF12.** Steady-state kinetics for sGalDH
**SF13.** Steady-state kinetics for sGalDH A113G oxidase activity
**SF14.** Steady-state kinetics for sGalDH A113G dehydrogenase activity
**SF15.** Steady-state kinetics for sGalDH G413N
**SF16.** Steady-state kinetics for jGULO
**SF17.** Steady-state kinetics for tGULO
**SF18.** The overall structure of vGalDH
**SF19.** Pre-steady-state kinetics of mGULO
**SF20.** UHPLC/HRMS analysis of the overnight conversion of L-galactono-1,4-lactone and L-gulono-1,4-lactone into L-ascorbic acid
**SF21.** Multiple sequence alignment of extant aldonolactone oxidoreductases
**SF22.** Electron density maps for the FAD prosthetic group in the vGALD crystal structures

**Supplementary Tables** **Pages 24-27**

**ST1**. Apparent melting temperatures (°C) of ancestral GalDHs and GULOs
**ST2.** Steady-state kinetics of sGalDH, jGULO and tGULO
**ST3.** Data collection and refinement statistics
**ST4.** Active-site residues
**ST5.** Primers list used for quick change experiments

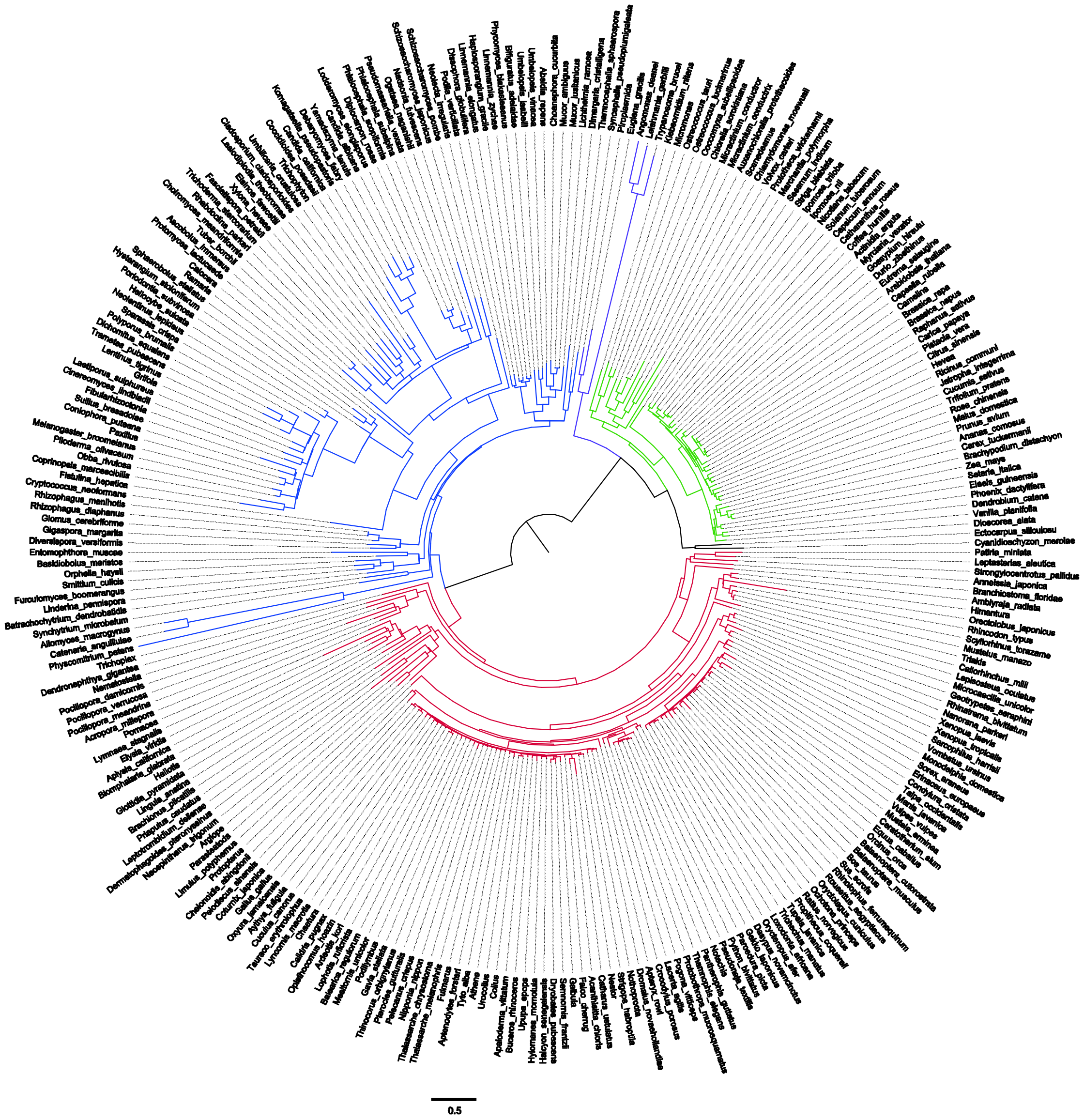


**Supplementary Figure 1. Fully annotated phylogeny of the eukaryotic aldonolactone oxidoreductases**. The tree was constructed in RAxML v8.2.10 using 500 rapid bootstraps and automated model selection. The employed multiple sequence alignment contained 268 sequences and 480 sites.Taxonomic groups (kingdom or phylum) are colored as follows: Euglenozoa (violet), Viridiplantae (green), Metazoa (red) and Fungi (blue). The species name is given for each taxa included in the dataset. The tree was prepared in Figtree v1.4.4. The accession codes to the protein sequences used for the phylogenetic analysis are provided as **Supplementary Data 1**.

>mGULO
MVHGHKGVKFQNWAKTYGCSPEMYFQPTSVEEIREVLDLARQQNKRVKVVGGGHSPSDIACTDGFMIHMGKMNRI
LKVDKEKKQVTVEAGILLADLNVELSKHGLALSNLGAVSDVTAAGVIGTGTHNTGIKHGILATQVVALTLLTASG
TILECSESSNAELFQAARVHLGCLGVILTVTFQCVPQFHLQETSFPSTLKEVLDNLDSHLKKSEYFRFLWFPHSE
NVSVIYQDHTNKPPSSSANWFWDYAIGFYLLEFLLWISTFLPCLVPWINRFFFWLLFTGKVENSNLSYKIFNYEC
RFKQHVQDWAIPIEKTKEALLELKAMLESNPKVVAHYPVEVRFTRGDDILLSPCFQRDSCYMNIIMYRPYGKDVP
RLDYWLAYETIMKKVGGRPHWAKAHTCTRKDFEKMYPAFQKFCAIREKLDPTGMFLNAYLEKVFY

**Overall posterior probability**: 0.99

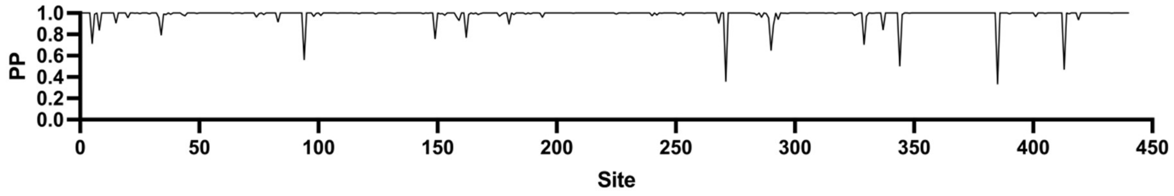


>tGULO
MVQGHGGYKFQNWAQTYGCSPELYFQPTSVEEIKEILDLARQRSKRVKVVGGGHSPSDIACTDDFMIHMNKMNKI
LKVDKEKKQVTVEAGILLSDLNEELSKHGLALSNLGAVSDVAAAGVIGTGTHNTGIQHGILATQVVALTLMTASG
EILECSESVNAELFQAARLHLGCLGVILTVTFQCVPAFHLQETQFPSTLKEVLDNLDSHLKKSEYFRFLWFPHTE
NVSVIYQDRTDKPPSSSANWFWDYAIGYYLLEFLLWISTFLPRLVPWINRFFFWLLYSGKVERVNLSYKVFNYEC
RFKQHVQDWAIPIEKTKEALLELKAWLESNPKVVAHFPVEVRFARGDDILLSPCYQRDSCYMNIIMYRPYGKDVP
RQQYWLAYENIMKKVGGRPHWAKAHTCTRKDFEKMYPGFQKFCSIREKLDPTGMFLNTYLEKVFY

**Overall posterior probability**: 0.97

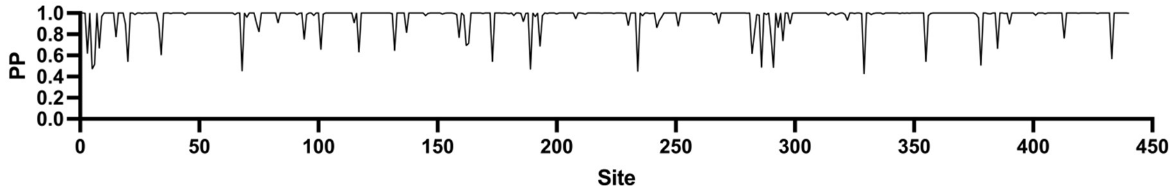

```
>jGULO
MVQGTEGYKFQNWAQTYSCQPELYFEPTSVEEIRQILELARQRSKRVKVVGCGHSPSDIACTDDYMIRMNKMNKI
LKVDKEKRQVTVEAGILLSDLNEELAKHGLALSNLGAVSDVTVAGVIGTGTHNTGIQHGILATQVVALTLMTASG
EILECSESVNKEIFQAARLHLGALGVILTVTFQCEPAFRLHQQQFPSTLTEVLDNLDTHLKKSEYFRFLWFPHTD
HVTVFYADRTDKPVQSTSSWFWDYAIGYYLLEFLLWISTFFPRLVPWINRFFFWLLYSAKVERVNRSDKVFNFEC
LFKQHVSDWAIPIEKTKAALLQLKEWLENNPDVRAHFPVEVRFVRADDILLSPCYQRDSCYINIIMYRPYGKDVP
RERYWAAYEDIMKKVGGRPHWAKAHSCTRKDFEKMYPAFQKFCSIREKLDPSGMFLNTYLEKVFF
```

**Overall posterior probability**: 0.94

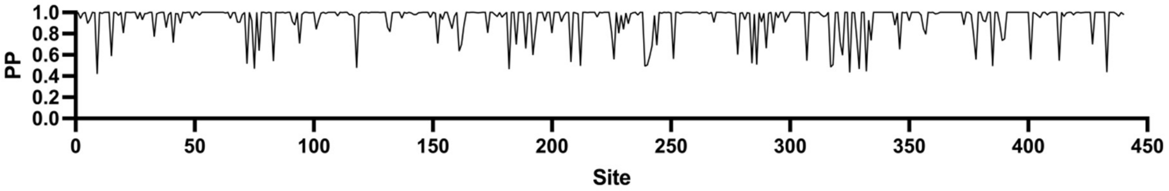


```
>cGULO
MATGMKGYKFTNWAKTYSCQPELYFEPTTTEEIRQILELARQHSKRVKVVGCGHSPSDIACTTDYMISMKKFNKV
LEVDKEKCQVTVQAGILLKDLNEELPKHGMALSNLGAVSDITVAGVISTGTHGTGVNYGILATHVVELTLMTASG
EILKCSREENKDIFLAALCGLGALGIILTVTIQCEPAFKLHQKQFPCTLDEVLDNLDTHLKSSEHFRFMWFPHTD
HVVVFHANRTDKPVQSTSSWFWDYLVGYYLLEFLLWISTFFPSLVPWINRFFFWLLYSQPKERVDRSDKVFNFEC
LFKQYVTEWAIPREKTAAVLLELKDWLESNPDFHAHFPVEVRFVRADDIYLSPCYQRDSCYINIIMYRPYGKDVP
REKYWAAYEDIMMKVGGRPHWAKAHKVTAKDFQKMYPKFEKFCSIREKLDPQGMFLNSYLERVFF
```

**Overall posterior probability**: 0.90

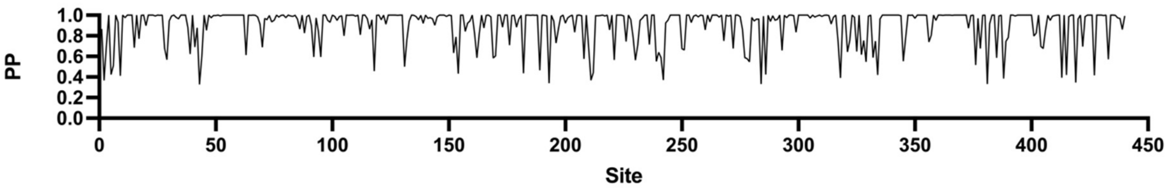

```
>metazoan ancestor GULO
MANNRNLDSDIMARGIKGYTFQNWAKTFSCQPELYFEPETTEEIRQILELARQHGKKVRVVGSGHSPSDIACTSG
YMISMKKLNKVLEVDKEKCQVTVQAGIMLKKLNEELPKHGLALSNLGSISDQTIAGAISTGTHGTGVNYGILATY
VVELELMTASGEILTCSREENPDIFRAALCSLGALGIILTVTLQCEPAFRLHQTQFPCTLDDVLDNLDSHIKSSE
HFRFMWFPHTDNVVVWHANRTDKPVPSKSSWFWDKLVGYHLLEFLLWISTFFPSLVPWINRFYFHLLYSQPKERV
DRSYKVFNFDCLFKQYVTEWAIPREKTAEVLRELKEWIEDNPNFHVHFPVEVRFVKADDIWLSPCYGRDTCYINI
IMYRPYGKDVPHEKYWNAYEDIMRSVGGRPHWAKAHKLTAKDLQKMYPKFDKFCEIRQKLDPNGMFLNDYLERHI
LGHSPQKTVNAKL
```

**Overall posterior probability**: 0.89

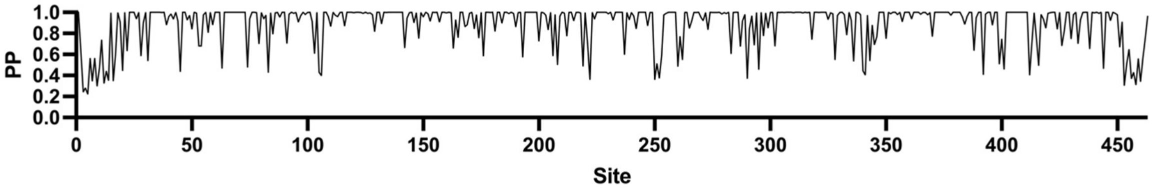


```
>fungal ancestor ALO
MSSSNNLDDELQARSLKNHTFQNWAKTFSCTPELYFEPETEEEIVQIIELARKHGKKVRVVGSGHSPSDIACTSG
YMINMDKLNRVLEVDKEKCTVTVQAGIRLHQLNEELEKHGLALSNLGSISDQSIAGAISTGTHGTGVNYGVLSTM
VVELTLITASGEILTCSREENPDLFRAALCSLGALGIIVQVTLQCEPAFRLRATQTPVKLDDVLDNLDSHIRSAE
HVRFWWFPHTDNCVVWRANRTDKPQPPKSSWFRDKLIGYHLYEFLLYISTFFPSLVPWINRFYFHLLFSKPTTVV
DKSYKVFNFDCLFKQYVNEWAIPWEKAPEALRQLKEWIEDEPNLRVHFPIEIRFVKADDIWLSPAYGRDTCYIGI
IMYRPYGKDVPHKKYWKAYEDIMRSFGGRPHWAKAHSLTPEDLQKMYPKFDDFCEIRQKLDPNGMFLNDYLERHI
LGDSPEKSVKAKL
```

**Overall posterior probability**: 0.88

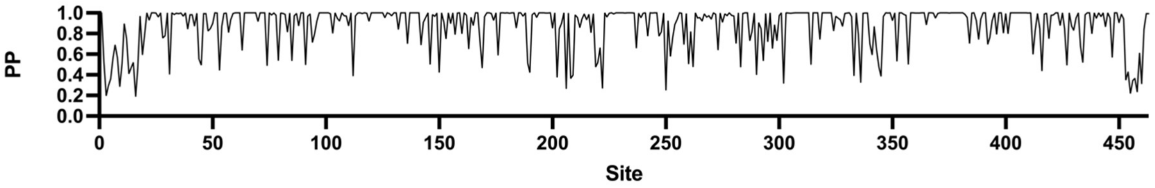

>Opisthokonta ancestor
MSSSNNLDEEIQARGIKNYTFQNWAKTFSCTPELYFEPETTEEIVQIIELARKHGKKVRVVGSGHSPSDIACTSG
YMISMDKLNKVLEVDKEKCTVTVQAGIRLHQLNEELPKHGLALSNLGSISDQSIAGAISTGTHGTGVNYGVLSTY
VVELELITASGEILTCSREENPDLFRAALCSLGALGIIVQVTLQCEPAFRLHATQFPATLDDVLDNLDSHIRSSE
HFRFWWFPHTDNCVVWRANRTDKPTPPKSSWFRDKLIGYHLLEFLLYISTFFPSLVPWINRFYFHLLYSKPTTVV
DRSYKVFNFDCLFKQYVNEWAIPREKAPEALRQLKEWIEDEPNFHVHFPIEVRFVKADDIWLSPAYGRDTCYIGI
IMYRPYGKDVPHKKYWKAYEDIMRSFGGRPHWAKAHSLTPEDLQKMYPKFDEFCEIRQKLDPNGMFLNDYLERHI
LGNSPEKSVKAKL

**Overall posterior probability**: 0.86

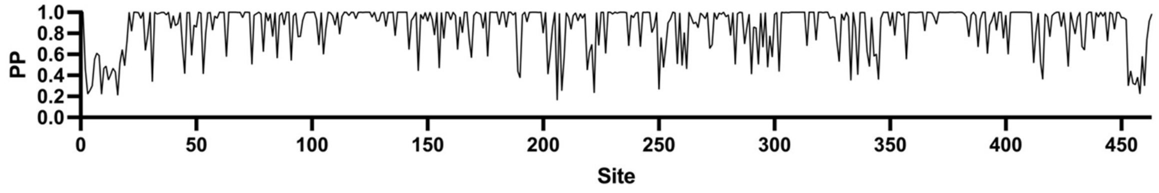

>sGalDH
MLRALLLRRSGGSLHHHRQTLNSPNSASNPSARGNPLRPLSSAGPRYSSAPPPPPPPEPPPPLPSSSLSDSEARK
YLSYTALLLGCGAATYYSFPRPENAKHKKAPIVRYAPLPEDVHTISNWSGTHEVKTRVFLQPESLEELEKVVKEA
HEKKQKIRPVGSGLSPNGIGLSRDGMVNLALMDKVLEVDKEKKQVTVQAGARVQQVVDALKPHGLTLQNFASIRE
QQIGGFIQVGAHGTGARLPPVDEQVVSMKLVTPAKGTIELSKEKDPELFYLARCGLGALGVVAEVTLQCVPRHKL
LEHTFVSNMKEVKKNHKKWLSENKHVRYMWIPYTDTVVVVQCNPLPEGKKSPKFKPKYSEDEKLQHVRDLYRETA
KKYRAEEVAAPSPSDTEPEISELSFTELRDKLLAVDPLNKDHVIKVNQAEAEFWKRSEGYRVGWSDEILGFDCGG
QQWVSEVAFPAGTLEKPSMKDLEYMEELMRLIDKEGIPAPAPIEQRWTASSKSPMSPASSSSPDSIFSWVGIIMY
LPTADEAQRKAITEAFRQYRKLTQTRLWDQYSAHEHWAKIEVPEDKEELAALQERLRKRFPGVDAFNKARRELDP
NRILSNDMIDKLFPSSETV

**Overall posterior probability**: full length 0.93, truncated (Δ120) 0.96

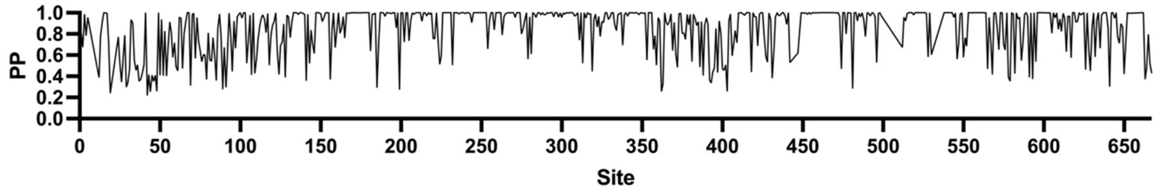

>vGalDH
MLRALLLRRSGGSLRHHAATLNSANAASNPKARGSAARGLASAGGRYTAAPPAPPPPEPPPPLPSSSLSASEAAK
YLAYGALLLGCGAATYYAFARPEDAKHKEAPVVRYAPAAEDEHTITNWSGTHAVRPKRFFQPESVEELEKIVKEA
HEKGQKIRPVGSGLSPNGLAFSEDGMVSLALMDKVLHVDKEKKQVTVQAGARVQQVVDALRPHGLTLQNFASISE
QQIGGFIQVGAHGTGARIPPVDEQVVSMKLVTPAKGTIELSEEKDPELFRLARCGLGALGVVTEVTLQCVPRHKL
LEHTFVATMKEVKKNHEKLLRENKHVRYMWIPYTDTVVVVTCNPLPEGKKAPKVKPQYSEDEKLQPLRNLLREAA
PPARAPEVAAPSSSETSPEVSGLSFTELRDALLAVDPLDTEWVKRVNQAEAEFWKRSEGYRVGWSDEILGFDCGG
QQWVSEVAFPAGTLEKPSAADLEYMEELMRLINKEGIPAPAPIEQRWTAGSSSPMSPAYSPSPDSVFSWVGIIMY
LPTEDEEQRKAITEAFRQYRKLCETRLWDKYGAAEHWAKIEVPEDPEELEALRERLRKRYPGVDKFNKARRELDP
KNILSNDMIDSLFPAAESA

**Overall posterior probability**: full length 0.81, truncated (Δ119) 0.85

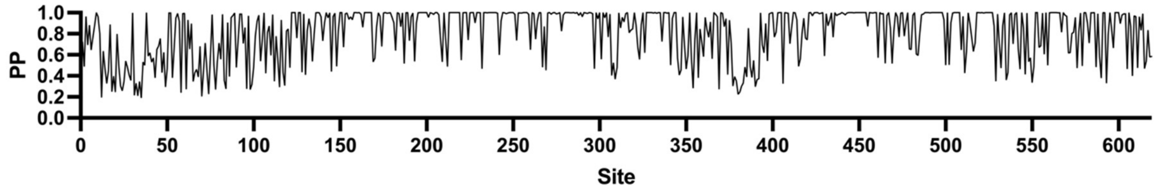


**Supplementary Figure 2. The amino acid sequences of the reconstructed aldonolactone oxidoreductases**. Graphs display the posterior probabilities at each of the reconstructed sites. The conserved mitochondrial target sequence is highlighted in red and was identified by aligning the sGalDH and vGalDH sequences with the sequence of the extant enzyme from *A. thaliana* (Uniprot: Q9SU56). The XR/YA cleavage site was used as trimming point to produce the proteins used for experimental characterization.

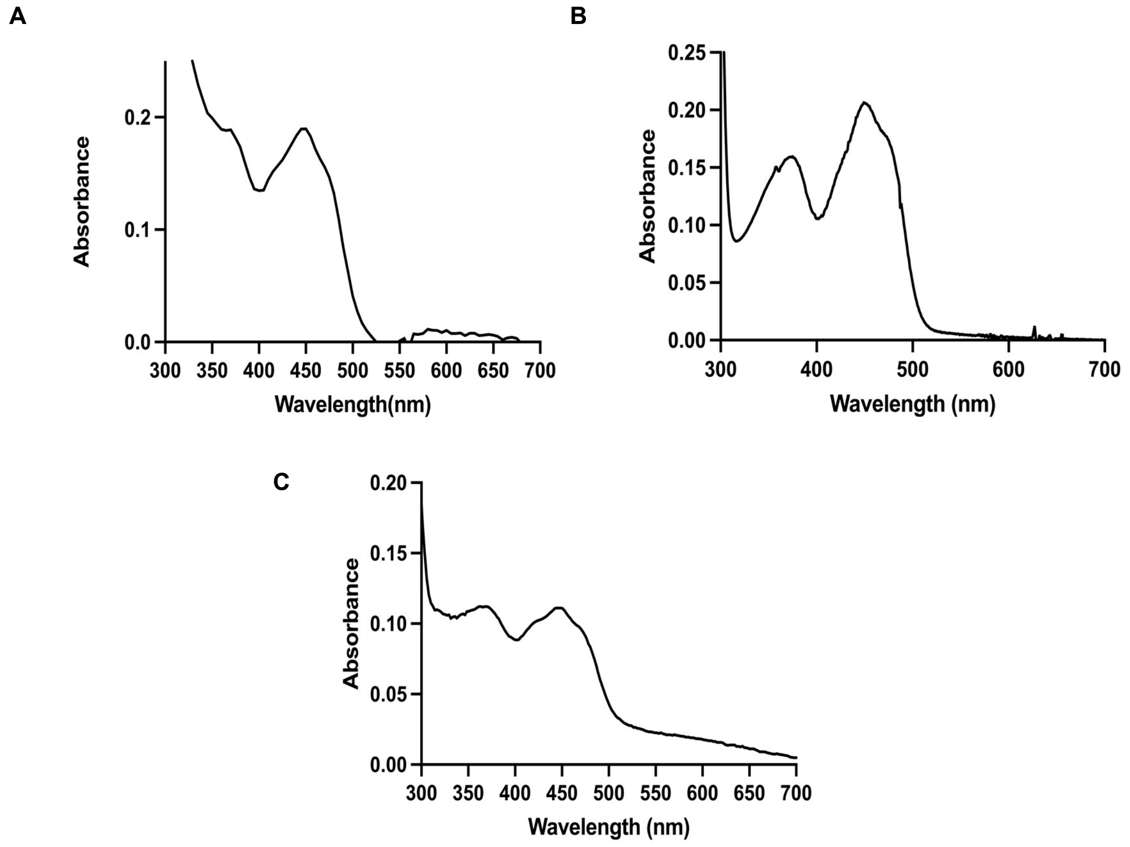


**Supplementary Figure 3. UV-Vis absorption spectra** of **A)** sGalDH, **B)** vGalDH, and **C)** mGULO.

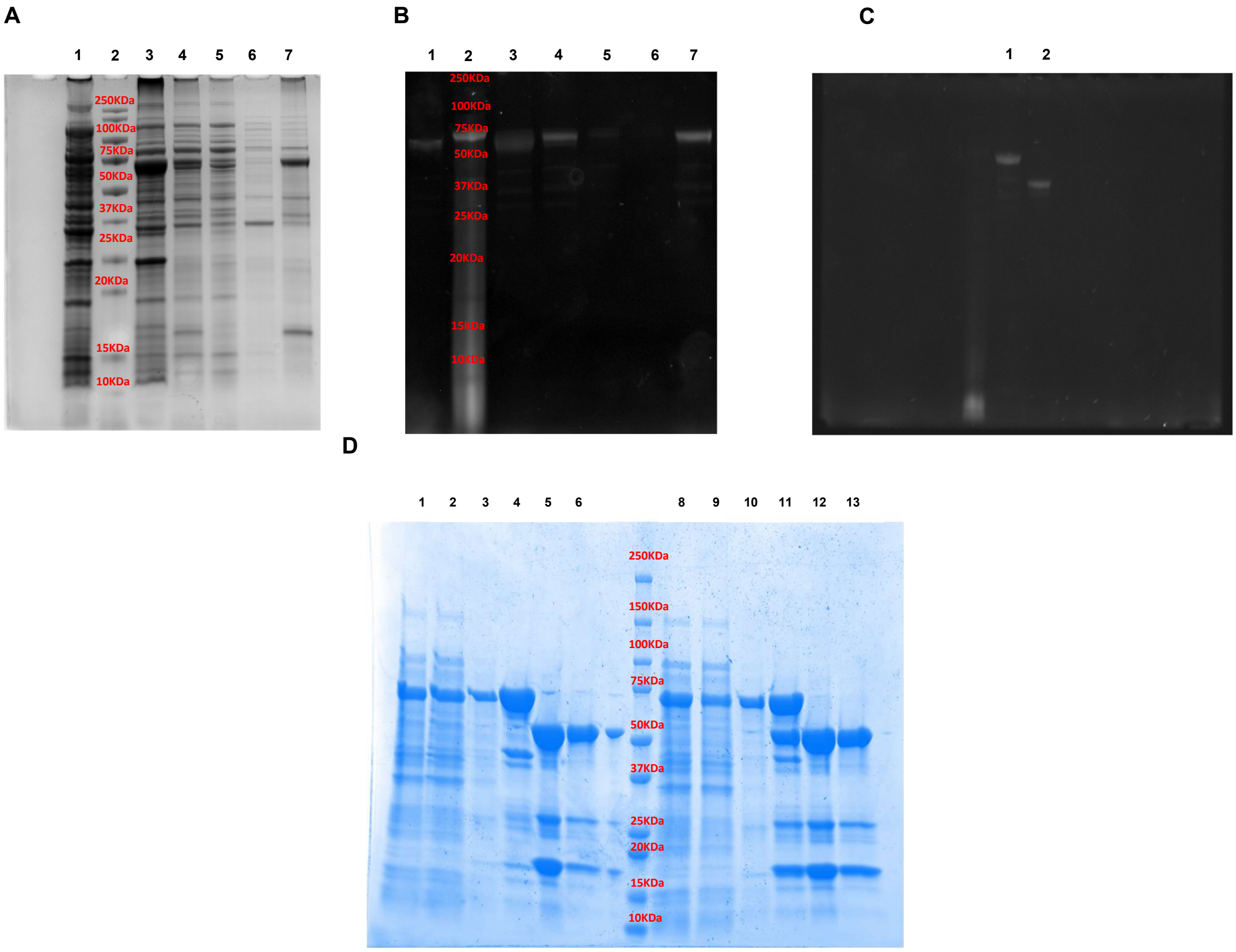


**Supplementary Figure 4. Uncropped SDS-PAGE gels of GalDHs and mGULO. A)** Purification steps of His-SUMO-mGULO. Lane 1: whole cell. Lane 2: molecular weight protein markers. Lane 3: pellet. Lane 4: after extraction. Lane 5: flow-through. Lane 6: washing step. Lane 7: Elution from the nickel-affinity column. **B)** Acetic acid staining under UV light of His-SUMO-mGULO. Lane 1: whole cell. Lane 2: markers. Lane 3: pellet. Lane 4: after extraction. Lane 5: flow-through. Lane 6: washing step. Lane 7: elution. **C)** mGULO before and after SUMO-cleavage with acetic acid staining under UV light. Lane 1: before SUMO cleavage. Lane 2: after SUMO cleavage. **D)** GalDH purification. Lane 1: Cell-free extract (sGalDH). Lane 2: flow-through (sGalDH). Lane 3: Washing step (sGalDH). Lane 4: Elution (sGalDH). Lane 5: After SUMO cleavage (sGalDH). Lane 6: flow-through of reverse nickel (sGalDH). Molecular weight protein markers. Lane 8: cell-free extract (vGalDH). Lane 9: flow-through (vGalDH). Lane 10: washing step (vGalDH). Lane 11: elution (vGalDH). Lane 12: after SUMO cleavage (vGalDH). Lane 13: flow-through of reverse nickel (vGalDH).

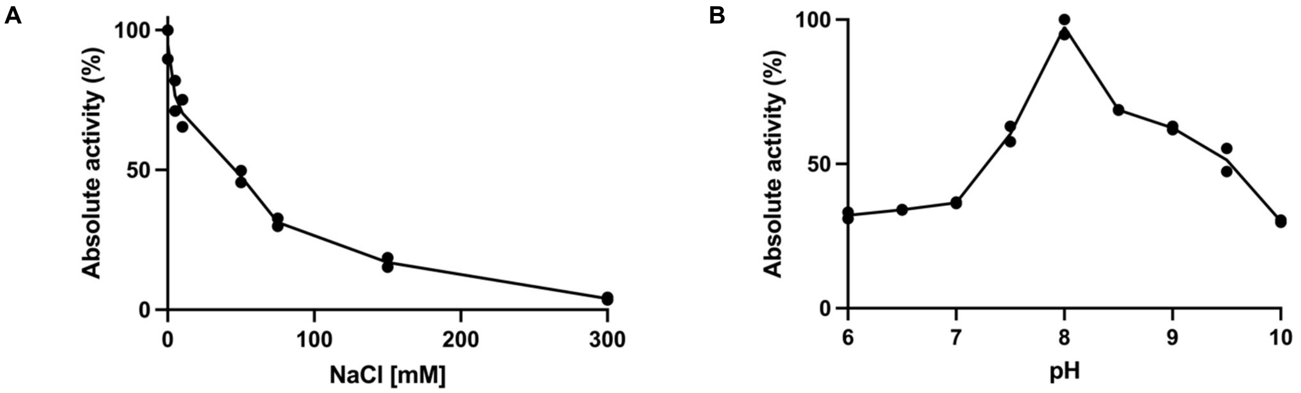


**Supplementary Figure 5**. **Effect of ionic strength and pH on vGalDH. A)** Salt inhibition effect using NaCl concentrations from 5 mM to 300 mM (pH 8). **B)** pH dependence from 6 to 10, using two different buffers (potassium phosphate and Tris-HCl). All reactions were performed in 50 mM buffer at 25 °C with 500 µM of L-galactono-1,4-lactone, 50 µM of equine cytochrome c, and 100 nM purified enzyme. n= 2 independent experiments, individually plotted as dots. **Source data** are provided.

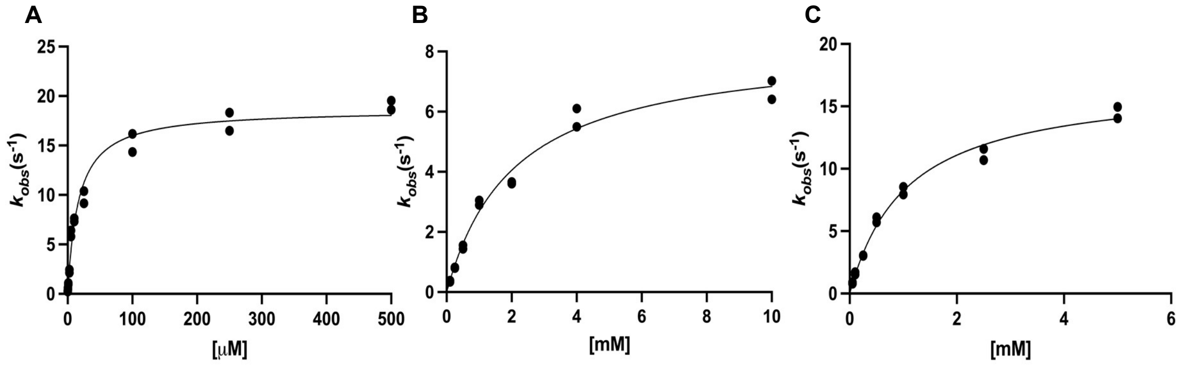


**Supplementary Figure 6**. **Steady-state kinetics for vGalDH**. **A)** vGalDH towards L-galactono-1,4-lactone. **B)** vGalDH towards L-gulono-1,4-lactone. **C)** vGalDH towards D-arabino-1,4-lactone. n= 2 independent experiments, individually plotted as dots. **Source data** are provided.

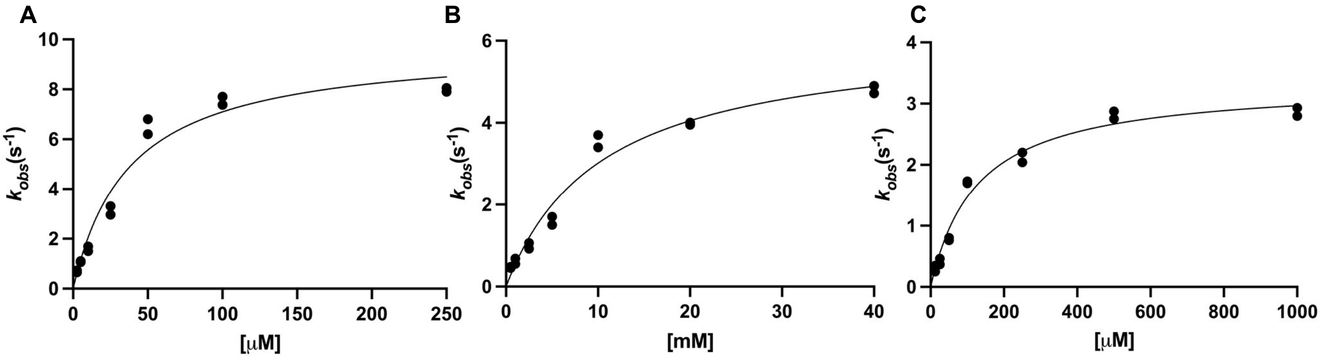


**Supplementary Figure 7**. **Steady-state kinetics for vGalDH A113G oxidase activity. A)** vGalDH A113G towards L-galactono-1,4-lactone. **B)** vGalDH A113G towards L-gulono-1,4-lactone. **C)** vGalDH A113G towards D-arabino-1,4-lactone. n= 2 independent experiments, individually plotted as dots. Recorded with an Oxygraph instrument by measuring oxygen depletion. **Source data** are provided.

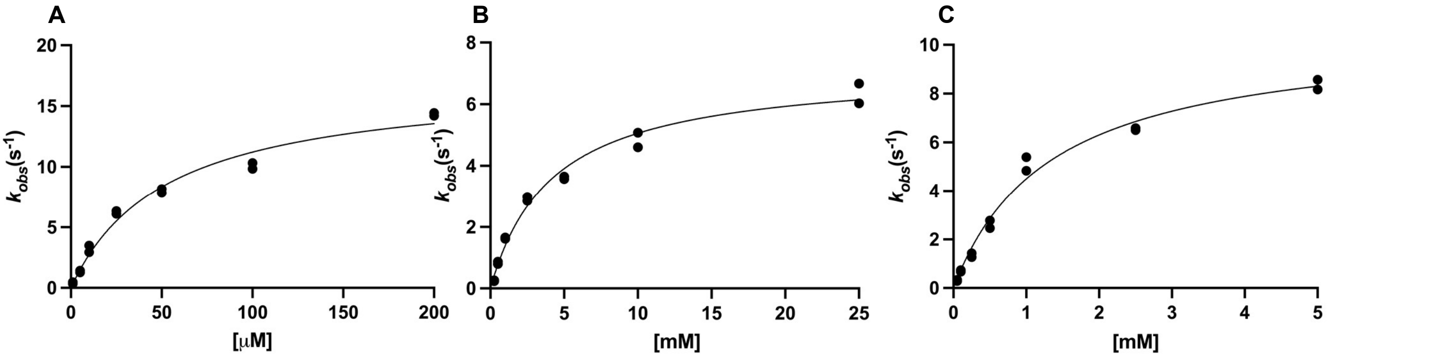


**Supplementary Figure 8**. **Steady-state kinetics for vGalDH A113G dehydrogenase activity. A)** vGalDH A113G towards L-galactono-1,4-lactone. **B)** vGalDH A113G towards L-gulono-1,4-lactone. **C)** vGalDH A113G towards D-arabino-1,4-lactone. n= 2 independent experiments, individually plotted as dots. **Source data** are provided.

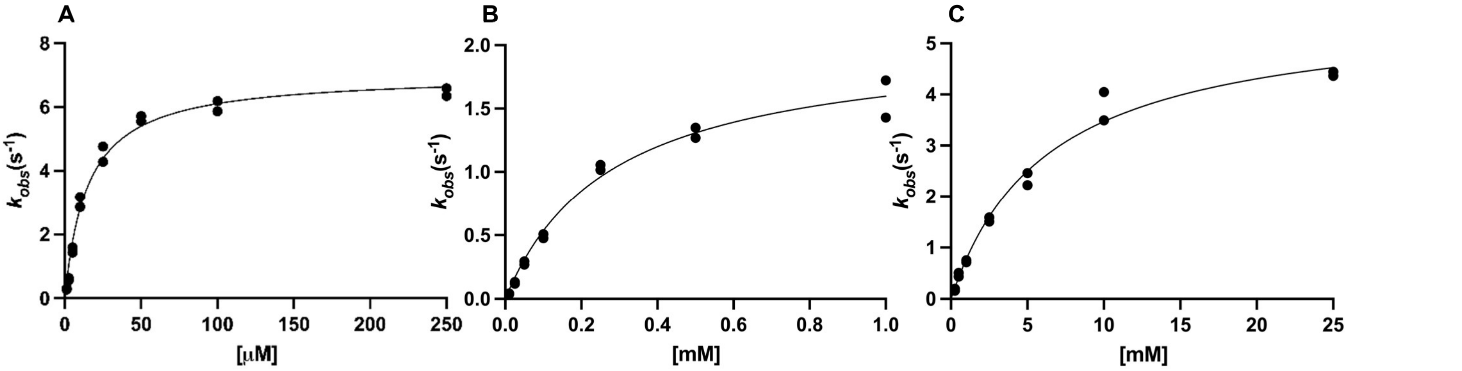


**Supplementary Figure 9**. **Steady-state kinetics for vGalDH G413N. A)** vGalDH G413N towards L-galactono-1,4-lactone. **B)** vGalDH G413N towards L-gulono-1,4-lactone. **C)** vGalDH G413N towards D-arabino-1,4-lactone. n= 2 independent experiments, individually plotted as dots. **Source data** are provided.

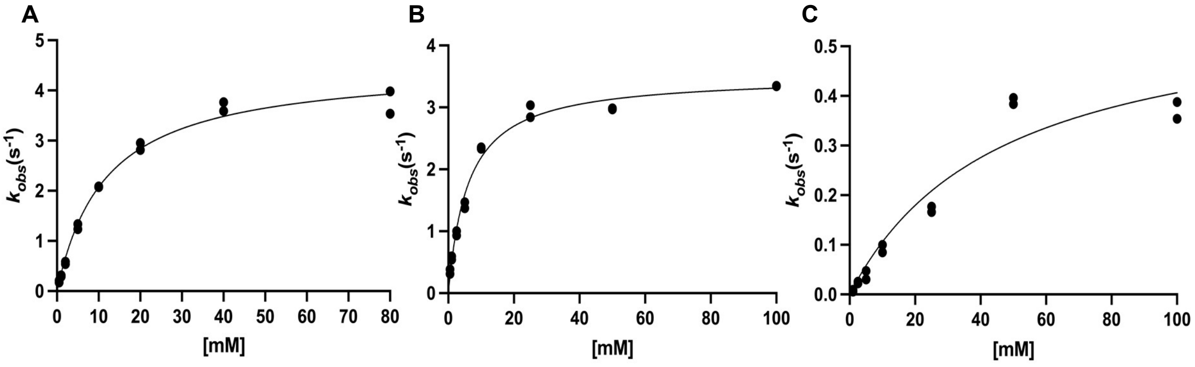


**Supplementary Figure 10**. **Steady-state kinetics for cGULO. A)** cGULO towards L-galactono-1,4-lactone. **B)** cGULO towards L-gulono-1,4-lactone. **C)** cGULO towards D-arabino-1,4-lactone. n= 2 independent experiments, individually plotted as dots. **Source data** are provided.

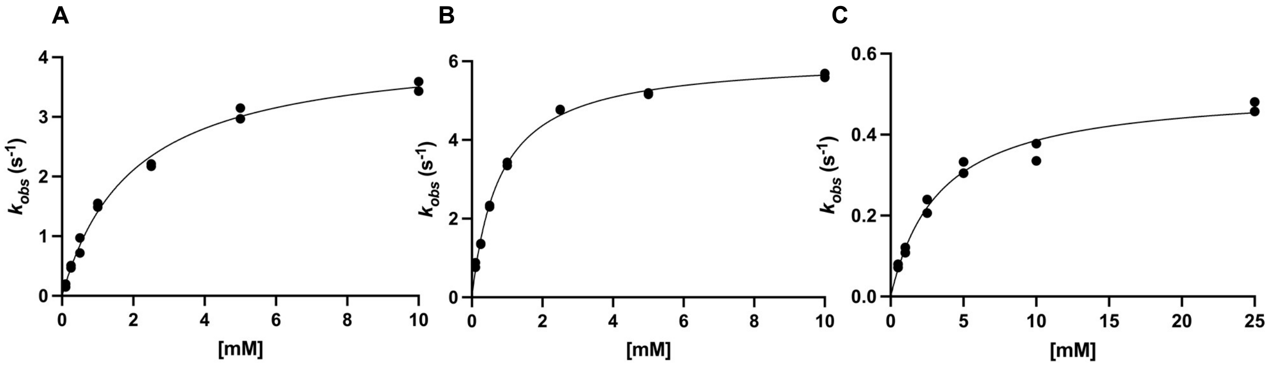


**Supplementary Figure 11**. **Steady-state kinetics for mGULO. A)** mGULO towards L-galactono-1,4-lactone. **B)** mGULO towards L-gulono-1,4-lactone. **C)** mGULO towards D-arabino-1,4-lactone. n= 2 independent experiments, individually plotted as dots. **Source data** are provided.

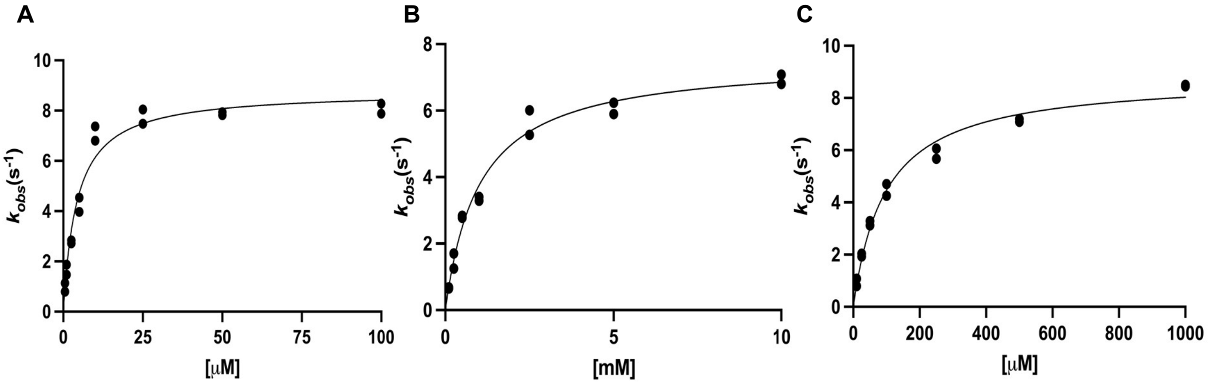


**Supplementary Figure 12**. **Steady-state kinetics for sGalDH**. **A)** sGalDH towards L-galactono-1,4-lactone. **B)** sGalDH towards L-gulono-1,4-lactone. **C)** sGalDH towards D-arabino-1,4-lactone. n= 2 independent experiments, individually plotted as dots. **Source data** are provided.

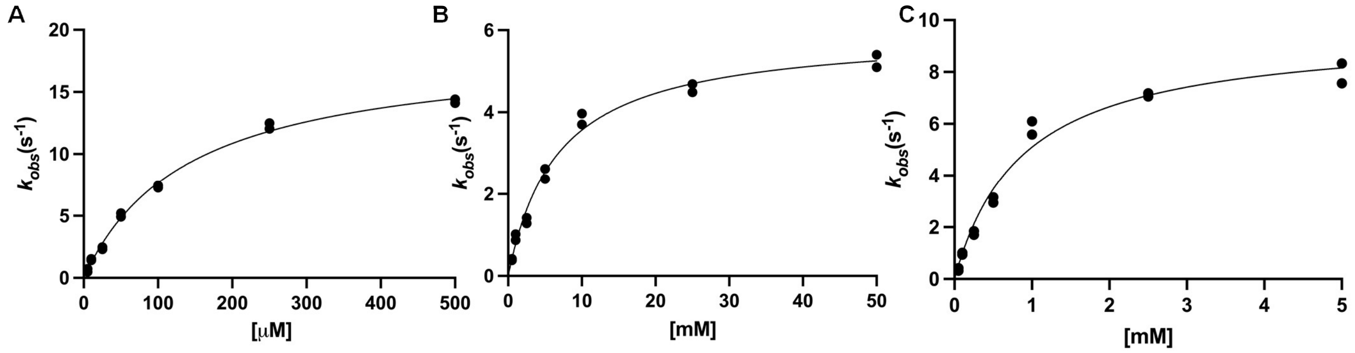


**Supplementary Figure 13**. **Steady-state kinetics for sGalDH A113G oxidase activity. A)** sGalDH A113G towards L-galactono-1,4-lactone. **B)** sGalDH A113G towards L-gulono-1,4-lactone. **C)** sGalDH A113G towards D-arabino-1,4-lactone. n= 2 independent experiments, individually plotted as dots. Recorded with an Oxygraph instrument by measuring oxygen depletion. **Source data** are provided.

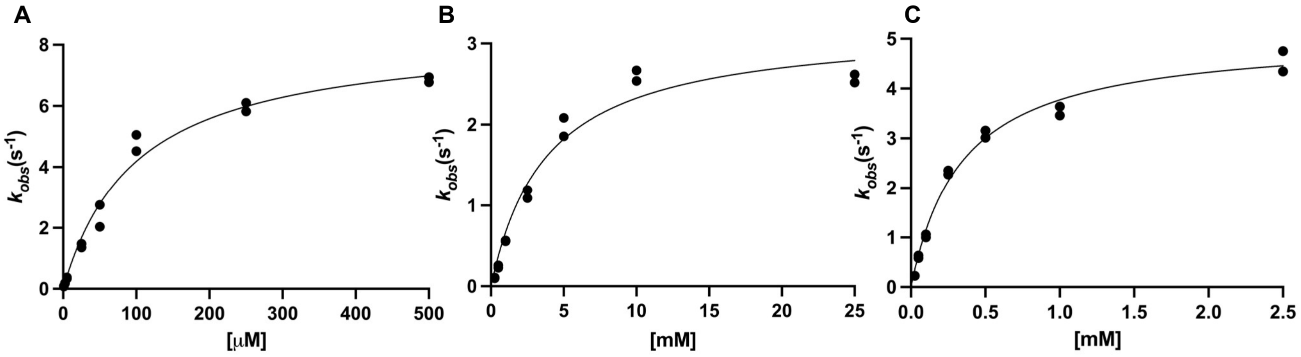


**Supplementary Figure 14**. **Steady-state kinetics for sGalDH A113G dehydrogenase activity. A)** sGalDH A113G towards L-galactono-1,4-lactone. **B)** sGalDH A113G towards L-gulono-1,4-lactone. **C)** sGalDH A113G towards D-arabino-1,4-lactone. n= 2 independent experiments, individually plotted as dots. **Source data** are provided.

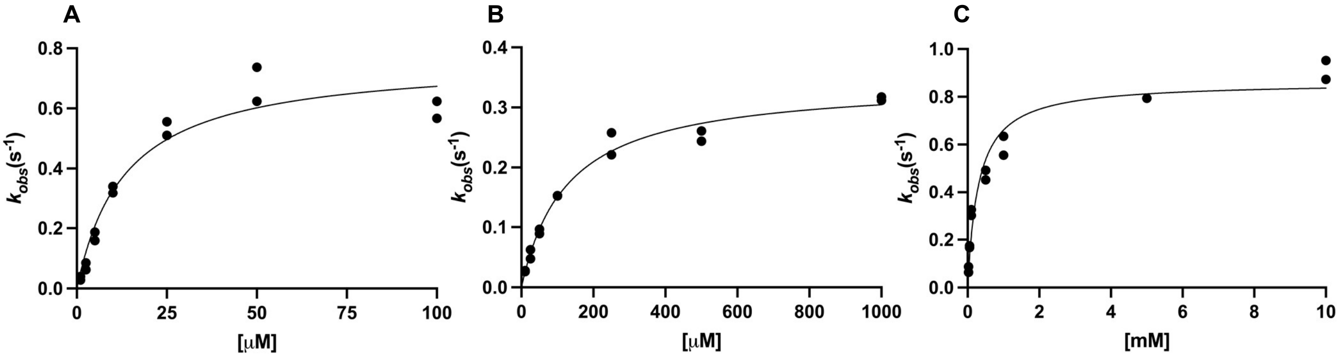


**Supplementary Figure 15**. **Steady-state kinetics for sGalDH G413N. A)** sGalDH G413N towards L-galactono-1,4-lactone. **B)** sGalDH G413N towards L-gulono-1,4-lactone. **C)** sGalDH G413N towards D-arabino-1,4-lactone. n= 2 independent experiments, individually plotted as dots. **Source data** are provided.

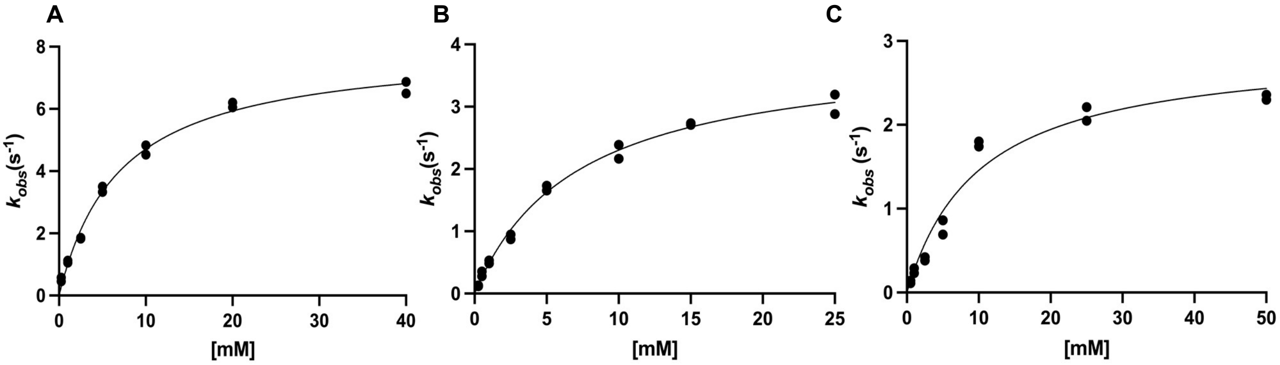


**Supplementary Figure 16**. **Steady-state kinetics for jGULO. A)** jGULO towards L-galactono-1,4-lactone. **B)** jGULO towards L-gulono-1,4-lactone. **C)** jGULO towards D-arabino-1,4-lactone. n= 2 independent experiments, individually plotted as dots. **Source data** are provided.

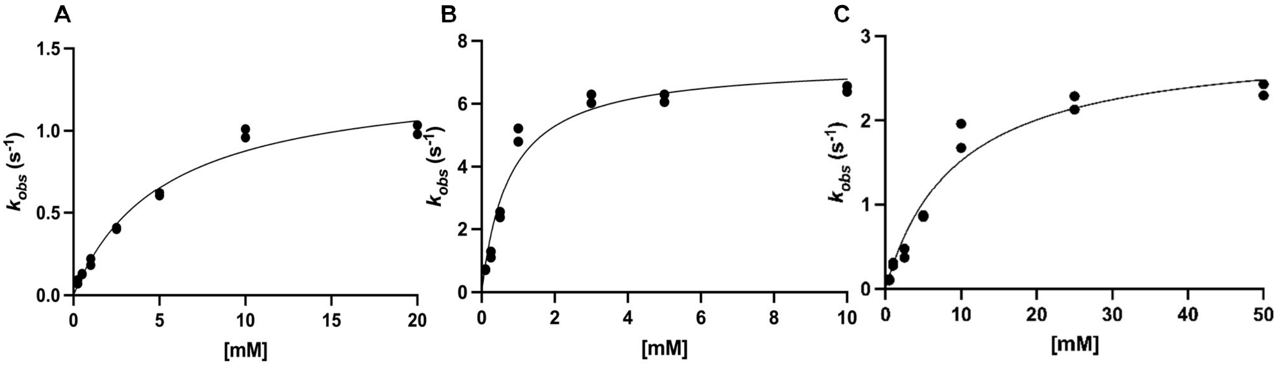


**Supplementary Figure 17**. **Steady-state kinetics for tGULO. A)** tGULO towards L-galactono-1,4-lactone. **B)** tGULO towards L-gulono-1,4-lactone. **C)** tGULO towards D-arabino-1,4-lactone. n= 2 independent experiments, individually plotted as dots. **Source data** are provided.

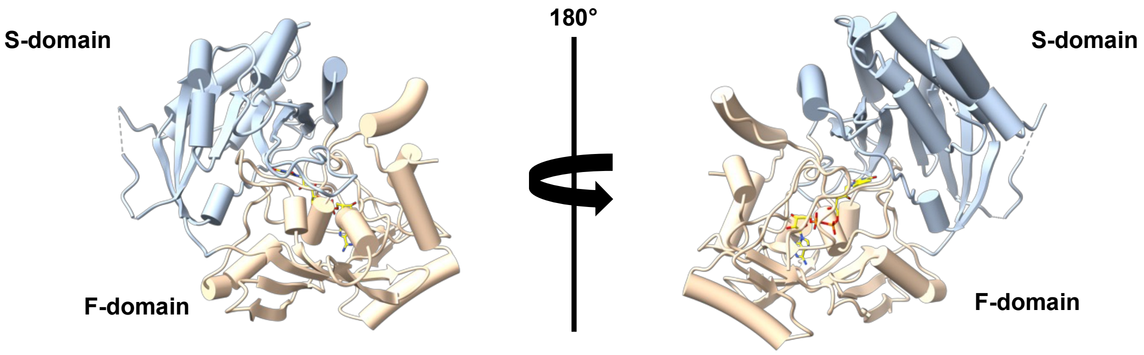


**Supplementary Figure 18. The overall structure of vGalDH.** The flavin domain is colored in wheat and labelled as F-domain, while the substrate domain is represented in light steel blue and labelled as S-domain. The flavin carbons are yellow. The enzyme was determined as monomeric by size exclusion chromatography experiments.

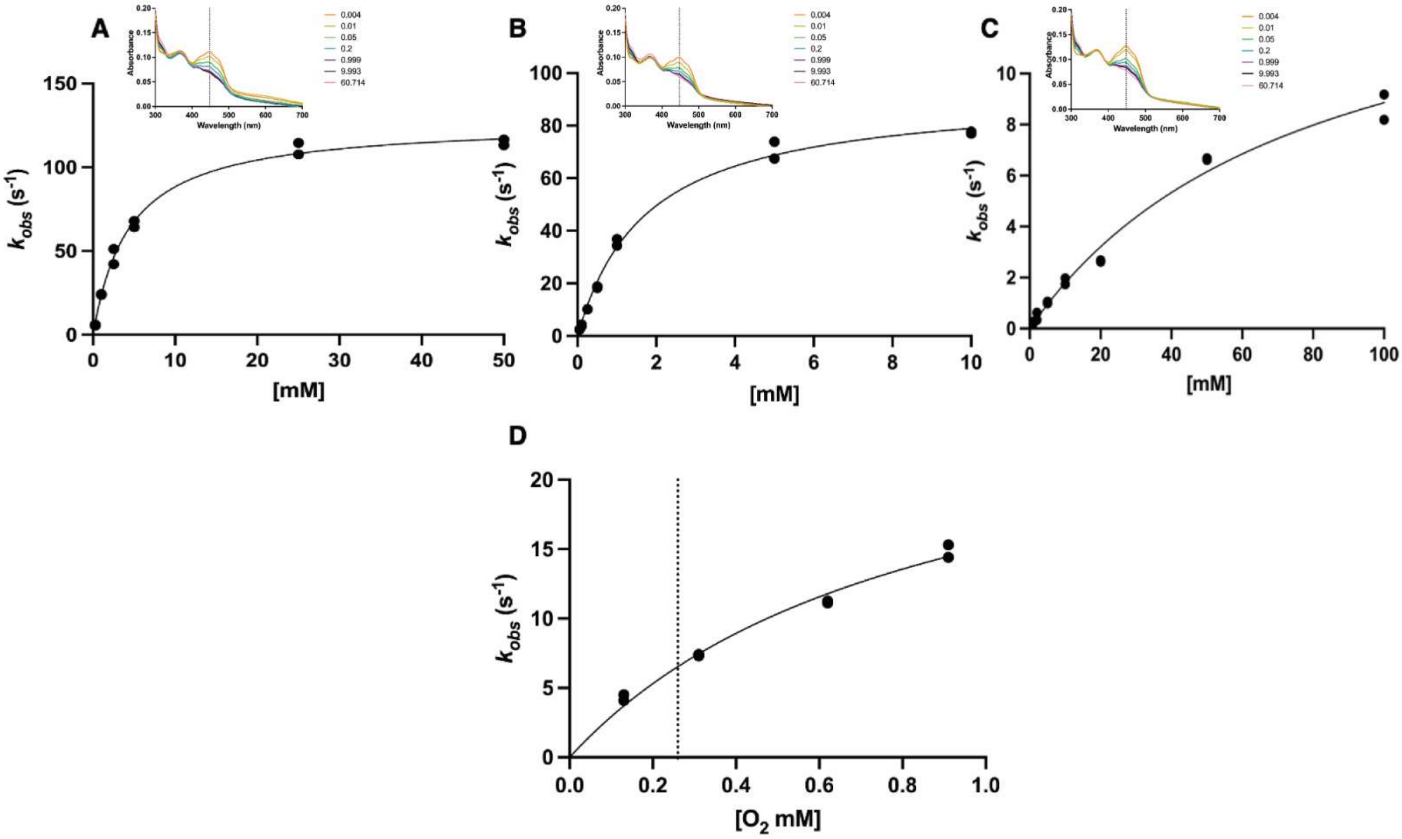


**Supplementary Figure 19. Pre-steady-state kinetics of mGULO.** Reduction rates ($k_{obs}$) plotted against different concentrations of **A**) L-galactono-1,4-lactone, **B**) L-gulono-1,4-lactone, and **C**) D-arabino-1,4-lactone. The insets show some spectra obtained during the reductive half-reaction using 1 mM substrate. **D**) The re-oxidation rates of mGULO at different oxygen concentrations. The dotted lines correspond to the atmospheric concentration of dioxygen (0.26 mM). For accurately determining rates of reduction or re-oxidation, absorption at 448 nm was monitored using the single wavelength detection mode of the stopped-flow instrument. n=2 independent experiments, individually plotted as dots. The experiments were performed with 5-10 µM enzyme concentrations (after mixing). **Source data** are provided.

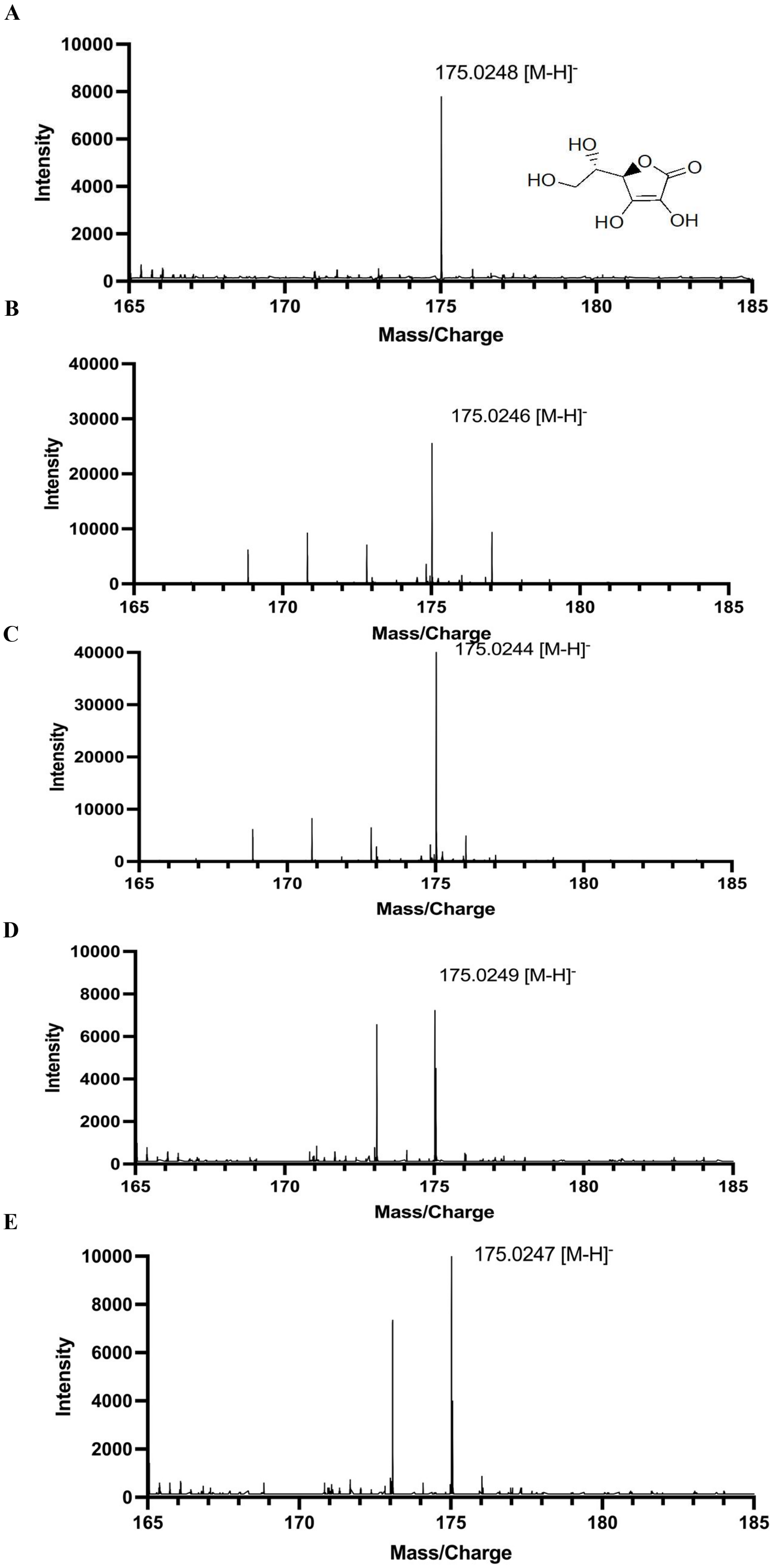
A
175.0248 [M-H]-
HO
HO
O
O
HO
OH
Intensity
Mass/Charge
B
175.0246 [M-H]-
Intensity
Mass/Charge
C
175.0244 [M-H]-
Intensity
Mass/Charge
D
175.0249 [M-H]-
Intensity
Mass/Charge
E
175.0247 [M-H]-
Intensity
Mass/Charge

**Supplementary Figure 20. UHPLC/HRMS analysis of the overnight conversion of L-galactono-1,4-lactone and L-gulono-1,4-lactone into L-ascorbic acid**. **A)** The extracted-ion chromatograms and ESI full-scan mass spectra of L-ascorbic acid recorded after the injection of the analytical standard. The theoretical mass of the $[M\text{-}H]^-$ molecular ion of L-ascorbic acid is 175.0248 Da. **B)** The extracted-ion chromatograms and ESI full-scan mass spectra of L-ascorbic acid after overnight conversion of L-gulono-1,4-lactone with vGalDH. It was detected with an error of -1.14 ppm in the analyses of the sample. **C)** The extracted-ion chromatograms and ESI full-scan mass spectra of L-ascorbic acid after overnight conversion of L-galactono-1,4-lactone with vGalDH. It was detected with an error of -2.29 ppm in the analyses of the sample. **D)** The extracted-ion chromatograms and ESI full-scan mass spectra of L-ascorbic acid after overnight conversion of L-gulono-1,4-lactone with mGULO. It was detected with an error of 0.57 ppm in the analyses of the sample. **E)** The extracted-ion chromatograms and ESI full-scan mass spectra of L-ascorbic acid after overnight conversion of L-galactono-1,4-lactone with mGULO. It was detected with an error of -0.57 ppm in the analyses of the sample.

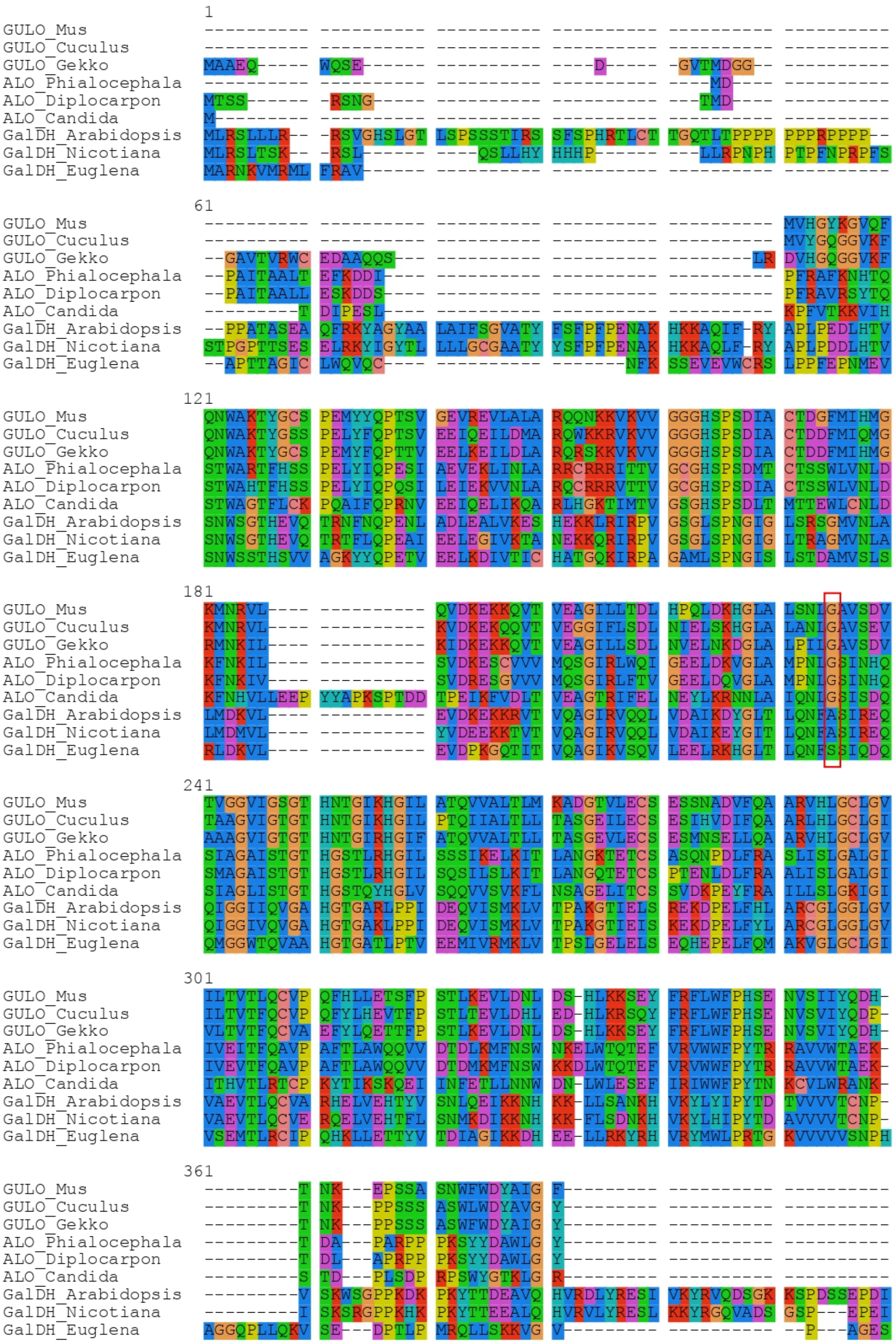

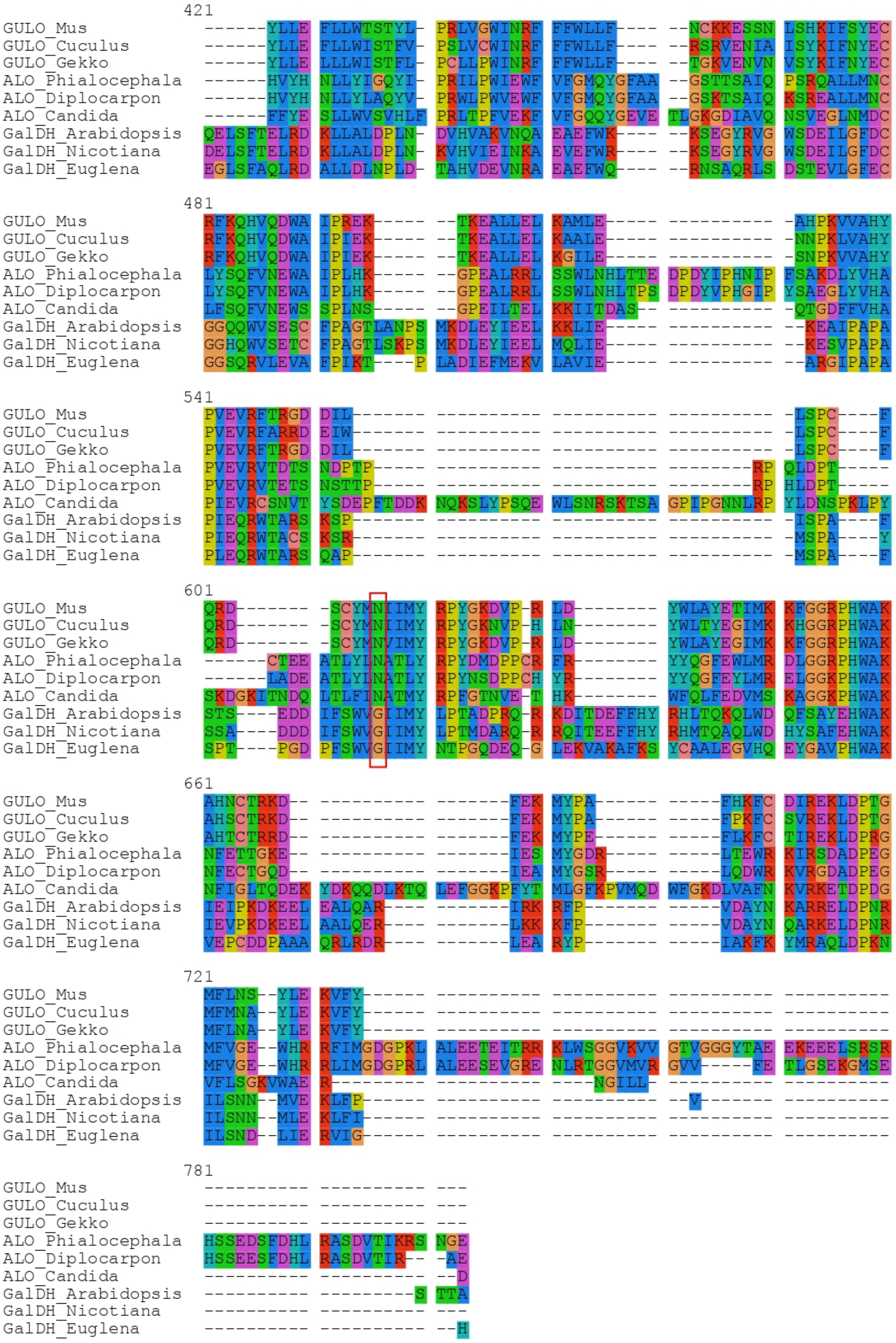


**Supplementary Figure 21. Multiple sequence alignment of representative extant aldonolactone oxidoreductases**. GULOs: P58710 (*M. musculus*), XP_053918765.1 (*C. canorus*), XP_015266653.1 (*G. japonicus*). ALOs: CZR68377.1 (*P. subalpina*), PBP25496.1 (*D. rosae*), XP_719313.1 (*C. albicans*). GalDHs: NP_190376.1 (*A. thaliana*), NP_001312725

(*N. tabacum*) and BAO94255.2 (*E. glacilis*). The alignment was constructed in MAFFTv7, and the image was prepared in SeaView. Amino acids are colored according to their chemical properties: KR, AFILMVW, NQST, HY, C, DE, P, G. In red boxes the two key positions identified in our work are highlighted.

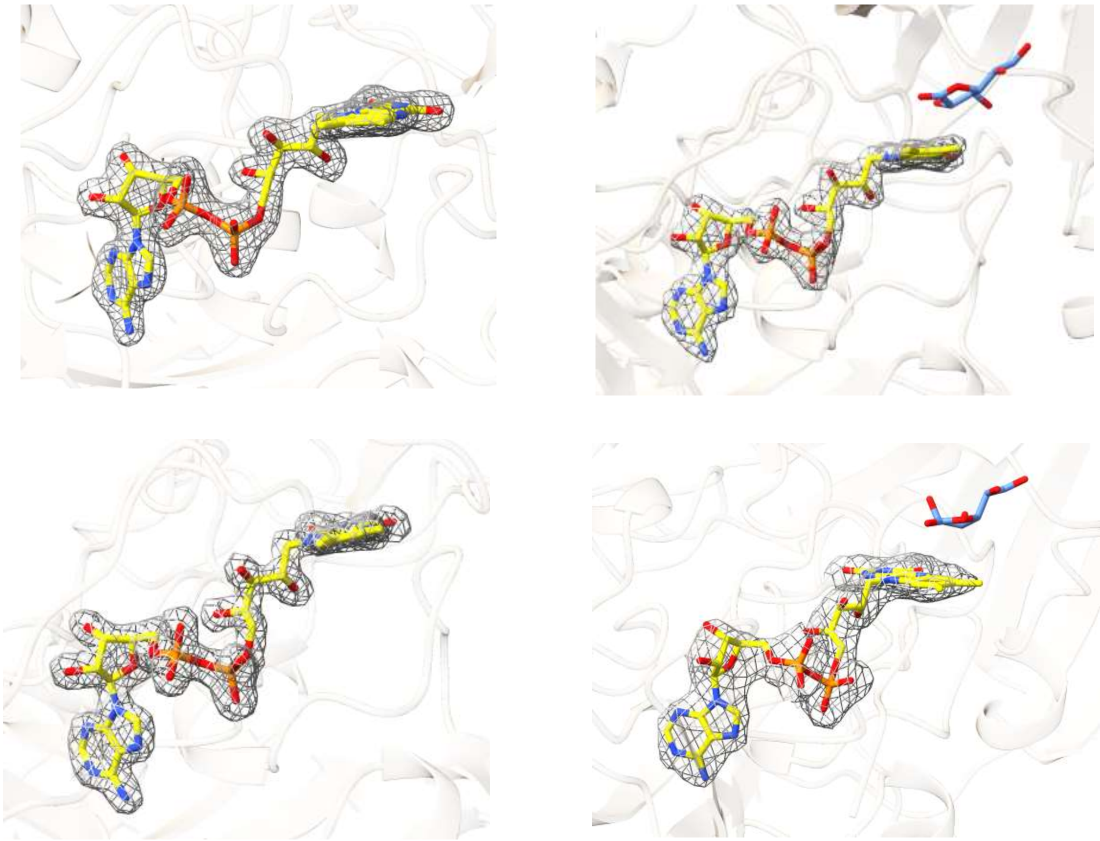


**Supplementary Figure 22. Electron density maps for the FAD prosthetic group in the vGALD crystal structures**. **A)** Wild-type enzyme, **B)** Wild-type in complex with L-galactono-1,4-lactone (carbons in cyan), **C)** A113G mutant, **D)** G413N mutant in complex with L-gulono-1,4-lactone (carbons in cyan). The weighted 2Fo-Fc maps are contoured at 1.2 $\sigma$.

**Supplementary Table 1. Apparent melting temperatures (°C) of ancestral GalDHs and GULOs**

| Enzyme | ***Tm*** **(°C)** |
|---|---|
| **sGalDHWt** | 68.7 |
| **vGalDHWt** | 86.8 |
| **sGalDHA113G** | 68.7 |
| **vGalDHA113G** | 82.9 |
| **sGalDHG413N** | 71.2 |
| **vGalDHG413N** | 86.9 |
| **cGULO** | 80 |
| **jGULO** | 80 |
| **tGULO** | 71.5 |
| **mGULO** | 66 |

Melting temperatures were measured by Differential Scanning Fluorimetry using a Tyco instrument (NanoTemper). A consecutive temperature gradient from 35 °C to 95 °C and 1 mg/ml protein were used.

**Supplementary Table 2. Steady-state kinetics of sGalDH, jGULO and tGULO**

| | Substrate | $K_M$ (mM) | $k_{cat}$ ($s^{-1}$) | $k_{cat}/K_M$ ($M^{-1}$ $s^{-1}$) |
|---|---|---|---|---|
| **sGalDH[a]** | L-galactono-1,4-lactone | 0.0043 ± 0.0008 | 8.7 ± 0.4 | $2x10^6$ |
| | L-gulono-1,4-lactone | 1.0 ± 0.1 | 7.6 ± 0.3 | $7.6x10^3$ |
| | D-arabino-1,4-lactone | 0.0974 ± 0.0134 | 8.8 ± 0.3 | $9x10^4$ |
| **sGalDH A113G (oxidase)[b]** | L-galactono-1,4-lactone | 0.142 ± 0.013 | 18.4±0.7 | $1.3x10^5$ |
| | L-gulono-1,4-lactone | 6.7 ± 0.800 | 5.9 ± 0.2 | $8.8x10^2$ |
| | D-arabino-1,4-lactone | 0.62 ± 0.17 | 9.6 ± 0.6 | $1.5x10^4$ |
| **sGalDH A113G (dehydrogenase)[a]** | L-galactono-1,4-lactone | 0.092 ± 0.017 | 8.4 ± 0.5 | $8.4x10^4$ |
| | L-gulono-1,4-lactone | 3.8 ± 0.9 | 3.2 ± 0.3 | $8.4x10^2$ |
| | D-arabino-1,4-lactone | 0.344 ± 0.043 | 5.1 ± 0.2 | $1.5x10^4$ |
| **sGalDH G413N[a]** | L-galactono-1,4-lactone | 0.012 ± 0.004 | 0.7 ± 0.1 | $5.8x10^4$ |
| | L-gulono-1,4-lactone | 0.091 ± 0.019 | 0.3 ± 0.02 | $3.3x10^3$ |
| | D-arabino-1,4-lactone | 0.3 ± 0.1 | 0.9 ± 0.1 | $3x10^3$ |
| | | | | |
| **jGULO[b]** | L-galactono-1,4-lactone | 7.1 ± 0.8 | 8.0 ± 0.3 | $1.1x10^3$ |
| | L-gulono-1,4-lactone | 6.9 ± 0.6 | 3.9 ± 1.3 | $5.6x10^2$ |
| | D-arabino-1,4-lactone | 10.7 ± 2.5 | 2.9 ± 0.3 | $2.7x10^2$ |
| **tGULO[b]** | L-galactono-1,4-lactone | 5.4 ± 1.2 | 1.3 ± 0.1 | $2.4x10^2$ |
| | L-gulono-1,4-lactone | 0.8 ± 0.2 | 7.3 ± 0.5 | $9.1x10^3$ |
| | D-arabino-1,4-lactone | 9.4 ± 0.9 | 2.9 ± 0.1 | $3.1x10^2$ |

[a]Measured at 25 °C in 50 mM potassium phosphate buffer pH 8 with 50 μM equine cytochrome c. Activities below 0.2 $s^{-1}$ were measured when oxygen was used in place of cytochrome c.
[b]Measured at 25 °C in 50 mM potassium phosphate buffer pH 8 by monitoring oxygen consumption.
**Supplementary Figures 12-17** and **Source Data** show the kinetics data.

## Supplementary Table 3. Data collection and refinement statistics

| **Diffraction Data** | **Wild type** | **Wild-type in complex with L-galactono-1,4-lactone** | **A113G variant** | **G413N variant in complex with L-gulono-1,4-lactone** |
|---|---|---|---|---|
| PDB code | 8QMY | 8QNB | 8QNC | 8QNR |
| Space group | $P2_12_12_1$ | $P2_1$ | $P2_1$ | $P2_1$ |
| Unit cell axes (Å) | 68.05, 98.02, 238.66 | 57.53, 69.96, 71.24 | 53.39, 69.66, 67.61 | 57.28, 70.03, 69.63 |
| Unit cell angles (°) | 90, 90, 90 | 90, 103.72, 90 | 90, 98.29, 90 | 90, 102.16, 90 |
| Resolution (Å) | 48.05-1.90 | 49.20-2.10 | 66.9-1.70 | 70.03-2.30 |
| $R_{merge}$ | 0.09 (1.734) | 0.091 (1.772) | 0.092 (1.336) | 0.029 (0.541) |
| $R_{pim}$ | 0.038 (0.702) | 0.043 (0.860) | 0.043 (0.618) | 0.029 (0.541) |
| $CC_{1/2}$ | 0.998 (0.562) | 0.999 (0.787) | 0.998 (0.593) | 0.999 (0.739) |
| Completeness (%) | 99.9 (99.8) | 98.3 (98.9) | 98.9 (99.4) | 92.4 (98.0) |
| Unique reflections | 126385 (6164) | 31671 (2611) | 53389 (2825) | 22213 (2314) |
| Multiplicity | 6.7 (6.2) | 5.3 (5.0) | 5.5 (5.5) | 1.4 (1.6) |
| Overall $I/\sigma(I)$ | 9.9 (1.1) | 13.9 (1.1) | 10.7 (1.3) | 8.9 (1.1) |
| Protein residues | 1414 | 474 | 486 | 465 |
| FAD molecules | 3 | 1 | 1 | 1 |
| Ligand | - | X8X | - | X8L |
| Water molecules | 572 | 131 | 295 | 76 |
| $R/R_{free}$ (%) | 19.5 /23.2 | 19.96 /25.43 | 17.87 /21.19 | 20.35 /28.31 |
| Rms bond length | 0.0086 | 0.0071 | 0.0110 | 0.0070 |
| Rms bond angle | 1.47 | 1.49 | 1.6784 | 1.49 |
| Ramachandran outliers (%) | 0.5 | 0.6 | 0.0 | 0.2 |
| Average B-factor ($Å^2$)/Occupancy | 42.79/1.00 | 50.03/1.00 | 27.00/1.00 | 67.66/1.00 |
| Protein | 42.79/1.00 | 50.34/1.00 | 27.25/1.00 | 67.66/1.00 |
| FAD | 59.7/1.00 | 34.13/1.00 | 33.6/1.00 | 43.9/1.00 |
| Ligand | - | 47.97/1.00 | - | 88.9/1.00 |
| Solvent | 42.96/1.00 | 48.42/1.00 | 34.82/1.00 | 57.11/1.00 |

Representative portions of the electron maps are shown in **Supplementary Figure 22**.

**Supplementary Table 4**. **Active-site residues**

| GalDH | | Opisthokontha ancestor | GULO | | | ALO | |
|---|---|---|---|---|---|---|---|
| vGalDH[a] | *A. thaliana*[b] | | Metazoa ancestor[a] | mGULO[a] | *M. musculus*[c] | Fungal ancestor[a] | *S. cerevisiae*[d] |
| Leu56 | Leu55 | His65 | His65 | His54 | His54 | His65 | His56 |
| Pro58 | Pro57 | Pro67 | Pro67 | Pro56 | Pro56 | Pro67 | Pro58 |
| Ala113 | Ala112 | Gly122 | Gly122 | Gly111 | Gly111 | Gly122 | Gly117 |
| Ser114 | Ser113 | Ala123 | Ser123 | Ala112 | Ala112 | Ser123 | Ser118 |
| Cys340 | Cys339 | Cys311 | Cys311 | Cys300 | Cys300 | Cys311 | Cys313 |
| Gln344 | Gln343 | Gln315 | Gln315 | Gln304 | Gln304 | Gln315 | Gln317 |
| Glu348 | Glu347 | Glu319 | Glu319 | Asp308 | Asp308 | Glu319 | Glu321 |
| Glu386 | Glu385 | Glu351 | Glu351 | Glu340 | Glu340 | Glu351 | Glu356 |
| Arg388 | Arg387 | Arg353 | Arg353 | Arg342 | Arg342 | Arg353 | Arg358 |
| Gly413 | Gly412 | Gly374 | Asn374 | Asn363 | Asn363 | Gly374 | Asn416 |
| Ile415 | Ile414 | Ile376 | Ile376 | Ile365 | Ile365 | Ile376 | Thr418 |
| Lys456 | Lys455 | Lys409 | Lys409 | Lys398 | Lys398 | Lys409 | Lys451 |

[a]The posterior probabilities were above 0.99 for all these sites (**Supplementary Figure 2**).
[b]The residue numbering refers to the mature protein after cleavage of the mitochondrial targeting sequence (the N-terminal 101 residues; UniProt Q9SU56).
[c]UniProt P58710.
[d]UniProt P54783.

**Supplementary Table 5. Primers list used for quick change experiments.**

| Primers name | Sequence |
|---|---|
| sGalDHA113G_FW | CAGAACTTTGGCTCTATTCGCGAACAGCAGATTGG |
| sGalDHA113G_RV | GCGAATAGAGCCAAAGTTCTGTAACGTCAAGCCGTG |
| vGalDHA113G_FW | CAAAACTTTGGCAGTATCAGTGAGCAGCAGATTGG |
| vGalDHA113G_RV | ACTGATACTGCCAAAGTTTTGGAGTGTCAGACCGT |
| sGalDHG413N_FW | AGCTGGGTGAATATCATCATGTACTTGCCTACGGC |
| sGalDHG413N_RV | CATGATGATATTCACCCAGCTAAAGATGGAATCGGG |
| vGalDHG413N_FW | AGTTGGGTAAATATCATTATGTACCTGCCGACCGAG |
| vGalDHG413N_RV | CATAATGATATTTACCCAACTGAATACGGAATCAG |
