# Supplementary material for: Structure, mechanism, and evolution of the last step in vitamin C biosynthesis
Source: Nat Commun. 2024 May 16;15:4158. doi: 10.1038/s41467-024-48410-1 (PMC11099136; doi:10.1038/s41467-024-48410-1)
Supplement: Supplementary file 3 — Description of Additional Supplementary Files [file 41467_2024_48410_MOESM3_ESM.pdf]

Description of Additional Supplementary Files

Supplementary Data 1. The accession codes to the protein sequences used for the phylogenetic analysis.
