# Supplementary material for: Structure, mechanism, and evolution of the last step in vitamin C biosynthesis
Source: Nat Commun. 2024 May 16;15:4158. doi: 10.1038/s41467-024-48410-1 (PMC11099136; doi:10.1038/s41467-024-48410-1)
Supplement: Supplementary file 4 — Supplementary Data 1 [file 41467_2024_48410_MOESM4_ESM.pdf]

Accession codes to the protein sequences used for the phylogenetic analysis

Metazoan		Fungi		Viridiplantae and Euglenozoa	
**Accession code**	**Organism**	**Accession code**	**Organism**	**Accession code**	**Organism**
XP_013393535.1	*Lingula anatina*	XP_719313.1	*Candida albicans SC5314*	CBJ29466.1	*Ectocarpus siliculosus*
RNA17712.1	*Brachionus plicatilis*	KAF0424573.1	*Gigaspora margarita*	OUS49455.1	*Ostreococcu s tauri*
XP_005103891.1	*Aplysia californica*	RHZ55450.1	*Diversispora versiformis*	XP_002501872.1	*Micromonas commoda*
ADI88511.1	*Haliotis discus*	PKY49459.1	*Rhizophagus manihotis*	XP_001417255.1	*Ostreococcu s lucimarinus CCE9901*
XP_025086131.1	*Pomacea canaliculata*	KNE68777.1	*Allomyces macrogynus ATCC 38327*	XP_005644279.1	*Coccomyxa subellipsoid ea C-169*
RDD39167.1	*Trichoplax sp. H2*	RIA90554.1	*Glomus cerebriforme*	PSC76821.1	*Micractiniu m conductor*
XP_014666894.1	*Priapulus caudatus*	ORX92044.1	*Basidiobolus meristosporus CBS 931.73*	GAX80918.1	*Chlamydom onas moewusii*
XP_019645195.1	*Orectolobus japonicus*	ORZ32170.1	*Catenaria anguillulae PL171*	EWM25741.1	*Piroplasmid a*
XP_035667342.1	*Branchiosto ma floridae*	TBU59901.1	*Dichomitus squalens*	PRW32883.1	*Chlorella sorokiniana*
ABO15547.1	*Triakis scyllium*	OJT12359.1	*Trametes pubescens*	XP_024359188.1	*Physcomitri um patens*
ABO15548.1	*Mustelus manazo*	XP_040762970.1	*Laetiporus sulphureus 93-53*	XP_010238743.1	*Brachypodi um distachyon*
XP_031758963.1	*Xenopus laevis*	KIY48528.1	*Fistulina hepatica ATCC 64428*	XP_004977506.1	*Setaria italica*
AGQ16462.1	*Himantura*	KAF8591766.1	*Ramaria rubella*	KAF3327167.1	*Carex littledalei*
XP_032881973.1	*Amblyraja radiata*	KAJ9077167.1	*Entomophthora muscae*	KAG0468274.1	*Vanilla planifolia*
XP_004682583.1	*Condylura cristata*	OLL26544.1	*Neolecta irregularis DAH-3*	KAF6261342.1	*Scenedesmu s sp. NREL 46B-D3*
XP_048457351.1	*Rhincodon typus*	RPA85402.1	*Ascobolus immersus RN42*	XP_002948012.1	*Volvox carteri f. nagariensis*
AGQ16461.1	*Protopterus annectens*	OAJ37206.1	*Batrachochytriu m dendrobatidis JEL423*	PTQ34691.1	*Marchantia polymorpha*
XP_015207781.1	*Lepisosteus oculatus*	XP_031026313.1	*Synchytrium microbalum*	XP_008808781.2	*Phoenix dactylifera*

XP_026571744.1	*Pseudonaja textilis*	CZR68377.1	*Phialocephala subalpina*	XP_010922174.1	*Elaeis guineensis*
XP_026527234.1	*Notechis scutatus*	XP_018701393.1	*Cordyceps fumosorosea ARSEF 2679*	OAY72857.1	*Ananas comosus*
XP_015680551.1	*Protobothrops mucrosquamatus*	OAX38422.1	*Cinereomyces_lindbladii*	XP_020703044.1	*Dendrobium catenatum*
XP_034277134.1	*Pantherophis guttatus*	RFU75829.1	*Trichoderma stercorarium*	XP_006290330.1	*Capsella rubella*
XP_032072268.1	*Thamnophis elegans*	Q90YK3.1	*Scyliorhinus torazame*	NP_190376.1	*Arabidopsis thaliana*
XP_020668255.1	*Pogona vitticeps*	RGB26044.1	*Rhizophagus sp. MUCL 43196*	XP_010515105.1	*Camelina sativa*
KAI8729503.1	*Biomphalaria glabrata*	NXR06194.1	*Semnornis frantzii*	XP_006404315.1	*Eutrema salsugineum*
OCT81467.1	*Xenopus tropicalis*	NXE16226.1	*Lophotis ruficrista*	RID58411.1	*Brassica rapa*
RUS84314.1	*Elysia viridis*	KAF8488900.1	*Poriodontia subvinosa*	XP_024948405.2	*Citrus sinensis*
XP_009051634.1	*Glottidia_ pyramidata*	KAF8507688.1	*Hysterangium stoloniferum*	BAO94255.2	*Euglena gracilis*
XP_029211884.2	*Acropora millepora*	KIJ47169.1	*Sphaerobolus stellatus SS14*	XP_018439846.1	*Raphanus sativus*
XP_020614299.1	*Pocillopora damicornis*	TFK29084.1	*Coprinopsis marcescibilis*	XP_005535453.1	*Cyanidioschyzon merolae strain 10D*
XP_027038466.1	*Pocillopora meandrina*	TFK47218.1	*Heliocybe sulcata*	EPY29186.1	*Angomonas deanei*
XP_020916795.1	*Pocillopora verrucosa*	XP_007773061.1	*Coniophora puteana RWD-64-598 SS2*	KAH7654930.1	*Dioscorea alata*
EDO44935.1	*Nematostella vectensis*	KZT24955.1	*Neolentinus lepideus HHB14362 ss-1*	KAH7654930.1	*Klebsormidium nitens*
XP_028394173.1	*Dendronephthya gigantea*	OCH88944.1	*Obba rivulosa*	XP_011773522.1	*Trypanosoma brucei gambiense DAL972*
XP_018423978.1	*Nanorana parkeri*	XP_027618010.1	*Sparassis crispa*	GET87693.1	*Leishmania gerbilli*
XP_030054536.1	*Microcaecilia unicolor*	EMD35022.1	*Piloderm olivaceum*	ONM19846.1	*Zea mays*
XP_025029314.1	*Python bivittatus*	OBZ74341.1	*Grifola frondosa*	XP_013706787.3	*Brassica napus*
XP_025897034.1	*Nothoprocta perdicaria*	KZP30052.1	*Fibularhizoctonia sp. CBS 109695*	XP_016731778.1	*Gossypium hirsutum*
XP_025922798.1	*Apteryx rowi*	RPD77041.1	*Lentinus tigrinus ALCF2SS1-7*	XP_011075395.2	*Sesamum indicum*
XP_033795170.1	*Geotrypetes seraphini*	RDX43699.1	*Polyporus brumalis*	XP_019187943.1	*Ipomoea nil*
XP_015266653.1	*Gekko japonicus*	KIJ18147.1	*Paxillus involutus ATCC 200175*	XP_024182616.1	*Rosa chinensis*
XP_025972375.1	*Dromaius novaehollandiae*	XP_568647.1	*Cryptococcus neoformans var. neoformans JEC21*	XP_021908679.1	*Carica papaya*

XP_015140704.1	*Gallus gallus*	KZT52131.1	*Calocera cornea HHB12733*	XP_031287265.1	*Pistacia vera*
XP_015715329.1	*Coturnix japonica*	TDL26994.1	*Suillus bresadolae*	XP_009306978.1	*Trypanosoma brucei*
XP_035178535.1	*Oxyura jamaicensis*	KAF9234587.1	*Melanogaster broomeanus*	XP_045807908.1	*Trifolium pratense*
XP_032040416.1	*Aythya fuligula*	XP_001523528.1	*Lodderomyces elongisporus*	XP_022766604.1	*Durio zibethinus*
KFV00817.1	*Tauraco erythrolophus*	XP_015468841.1	*Debaryomyces fabryi*	XP_021830036.1	*Prunus avium*
XP_053918765.1	*Cuculus canorus*	XP_002491085.1	*Komagataella pseudopastoris*	NP_001292704.1	*Cucumis sativus*
XP_010206806.1	*Colius striatus*	XP_006684519.1	*Yamadazyma tenuis ATCC 10573*	XP_031103303.1	*Ipomoea triloba*
XP_009871625.1	*Apaloderma vittatum*	ODQ68280.1	*Nadsonia fulvescens var. elongata DSM 6958*	NP_001312725.1	*Nicotiana tabacum*
NXI41105.1	*Galbula dea*	XP_036608783.1	*Lymnaea stagnalis*	XP_027176145.1	*Coffea humilis*
XP_054036133.1	*Dryobates pubescens*	KAF9119950.1	*Phycomyces blakesleeanus*	KAI5682742.1	*Catharanthus roseus*
XP_010141907.1	*Buceros rhinoceros silvestris*	KAF8945190.1	*Haplosporangium gracile*	KAF3635607.1	*Capsicum annuum*
NXD88061.1	*Halcyon senegalensis*	OMH86159.1	*Orphella haysii*	PSC76820.1	*Micractinium conductrix*
NXG69488.1	*Hylomanes momotula*	RKP09805.1	*Thamnocephalis sphaerospora*	XP_011401503.1	*Auxenochlorella protothecoides*
XP_042658980.1	*Tyto alba*	GAN02740.1	*Mucor ambiguus*	NP_001315785.1	*Malus domestica*
XP_026702137.1	*Athene cunicularia*	ORZ06936.1	*Absidia repens*	AKA45052.1	*Actinidia arguta*
XP_009078084.1	*Acanthisitta chloris*	CDS11481.1	*Lichtheimia ramosa*	KAF2289306.1	*Hevea brasiliensis*
XP_032911815.1	*Catharus ustulatus*	KAF9214115.1	*Podila verticillata*	XP_002531501.2	*Ricinus communis*
NXN92229.1	*Upupa epops*	KAF9296168.1	*Linnemannia elongata*	XP_012085977.1	*Jatropha integerrima*
XP_021135540.1	*Thalassarche chrysostoma*	OBZ89695.1	*Choanephora cucurbitarum*	CAA0837425.1	*Striga bilabiata*
XP_009936988.1	*Opisthocomus hoazin*	XP_002171556.1	*Schizosaccharomyces japonicus yFS275*	UNE55669.1	*Myrciaria vexator*

XP_010183407.1	*Mesitornis unicolor*	NP_593526.1	*Schizosaccharo myces pombe*	QDZ21362.1	*Prototheca wickerhamii*
XP_030346539.1	*Strigops habroptila*	XP_040725543.1	*Protomyces lactucae-debilis*		
NXL53322.1	*Podilymbus podiceps*	KAG0684435.1	*Candida californica*		
XP_010310844.1	*Balearica regulorum gibbericeps*	PUU83679.1	*Tuber borchii*		
XP_010012223.1	*Nestor notabilis*	XP_018185283.1	*Xylona heveae TC161*		
XP_010081542.1	*Pterocles gutturalis*	RPB06168.1	*Choiromyces meandriformis*		
XP_009465719.1	*Nipponia nippon*	PBP25496.1	*Diplocarpon rosae*		
NXP09293.1	*Thinocorus orbignyianus*	XP_040718238.1	*Pseudomassarie lla vexata*		
XP_014818284.1	*Calidris pugnax*	KAF3147938.1	*Umbilicaria crustulosa*		
XP_009489099.1	*Pelecanus crispus*	KMM72616.1	*Coccidioides posadasii RMSCC 3488*		
XP_009571134.1	*Fulmarus glacialis*	XP_018077959.1	*Phialocephala scopiformis*		
XP_027667359.1	*Falco cherrug*	KZL80272.1	*Ogataea naganishii*		
KFV56815.1	*Gavia stellata*	XP_003232226.1	*Trichophyton rubrum CBS 118892*		
XP_009273398.1	*Aptenodytes forsteri*	KAA6412188.1	*Fasciatispora_p etrakii*		
XP_032657598.1	*Chelonoidis abingdonii*	KAF4548658.1	*Elsinoe fawcettii*		
XP_010000521.1	*Chaetura pelagica*	XP_035374034.1	*Lasiodiplodia theobromae*		
NP_001303983.1	*Pelodiscus sinensis*	RKP27322.1	*Syncephalis pseudoplumigal eata*		
NXW51606.1	*Lyncornis macrotis*	KAJ1993902.1	*Dimargaris cristalligena*		
XP_007475977.1	*Monodelphis domestica*	KAF2431407.1	*Cladosporium cladosporioides*		
XP_036880511.1	*Manis javanica*	PVU92000.1	*Furculomyces boomerangus*		
XP_023351196.1	*Sarcophilus harrisii*	XP_040743504.1	*Linderina pennispora*		
XP_027706054.1	*Vombatus ursinus*	OMJ27386.1	*Smittium culicis*		
XP_016041119.1	*Erinaceus europaeus*	KAG2187216.1	*Umbelopsis vinacea*		
XP_054999526.1	*Sorex araneus*	KAG0310769.1	*Dissophora globulifera*		
XP_007192798.2	*Balaenoptera acutorostrata*	KAG2183308.1	*Umbelopsis isabellina*		
XP_033285702.1	*Orcinus orca*	KAF1804850.1	*Mucor lusitanicus*		
XP_032188235.1	*Mustela erminea*	OZJ03764.1	*Bifiguratus adelaidae*		
XP_025863664.1	*Vulpes vulpes*	KAF9909777.1	*Linnemannia zychae*		
XP_005607755.1	*Equus caballus*				
XP_014645409.1	*Ceratotheriu m simum simum*				

XP_016000110.2	*Rousettus aegyptiacus*
XP_032990521.1	*Rhinolophus ferrumequinum*
XP_008247496.1	*Oryctolagus cuniculus*
XP_058526467.1	*Ochotona princeps*
NP_071556.2	*Rattus norvegicus*
XP_006153286.1	*Tupaia javanica*
XP_012518430.1	*Propithecus coquereli*
XP_004454215.2	*Dasypus novemcinctus*
XP_003412410.1	*Loxodonta africana*
XP_012412689.2	*Trichechus manatus latirostris*
XP_042636607.1	*Orycteropus afer afer*
NP_001029215.1	*Bos taurus*
NP_001123420.1	*Sus scrofa*
XP_040062299.2	*Neospintharus trigonum*
XP_015913395.1	*Parasteatoda tepidariorum*
XP_019392806.1	*Crocodylus porosus*
XP_027193755.1	*Dermatophagoides pteronyssinus*
XP_013788034.1	*Limulus polyphemus*
RWS27066.1	*Leptotrombidium deliense*
XP_033100041.1	*Anneissia japonica*
XP_030837061.1	*Strongylocentrotus purpuratus*
XP_038052678.1	*Patiria miniata*
XP_022110599.1	*Leptasterias aleutica*
XP_036710021.1	*Balaenoptera musculus*
XP_060115885.1	*Paroedur picta*
XP_042189667.1	*Callorhinchus milii*
NXE29115.1	*Ardeotis kori*
NXX86585.1	*Urocolius indicus*
NXU27825.1	*Thalassarche melanophris*
XP_029452079.1	*Rhinatrema bivittatum*
XP_037382605.1	*Talpa occidentalis*
XP_055936822.1	*Argiope bruennichi*
