# Supplementary material for: Structure, mechanism, and evolution of the last step in vitamin C biosynthesis
Source: Nat Commun. 2024 May 16;15:4158. doi: 10.1038/s41467-024-48410-1 (PMC11099136; doi:10.1038/s41467-024-48410-1)
Supplement: Supplementary file 5 — Reporting Summary [file 41467_2024_48410_MOESM5_ESM.pdf]

nature portfolio

Corresponding author(s): M.L. Mascotti, M.W. Fraaije, A. Mattevi

Last updated by author(s): Apr 19, 2024

Reporting Summary

Nature Portfolio wishes to improve the reproducibility of the work that we publish. This form provides structure for consistency and transparency in reporting. For further information on Nature Portfolio policies, see our Editorial Policies and the Editorial Policy Checklist.

Statistics

For all statistical analyses, confirm that the following items are present in the figure legend, table legend, main text, or Methods section.

n/a	Confirmed	
☐	☒	The exact sample size (n) for each experimental group/condition, given as a discrete number and unit of measurement
☐	☒	A statement on whether measurements were taken from distinct samples or whether the same sample was measured repeatedly
☒	☐	The statistical test(s) used AND whether they are one- or two-sided<br>*Only common tests should be described solely by name; describe more complex techniques in the Methods section.*
☒	☐	A description of all covariates tested
☒	☐	A description of any assumptions or corrections, such as tests of normality and adjustment for multiple comparisons
☒	☐	A full description of the statistical parameters including central tendency (e.g. means) or other basic estimates (e.g. regression coefficient) AND variation (e.g. standard deviation) or associated estimates of uncertainty (e.g. confidence intervals)
☒	☐	For null hypothesis testing, the test statistic (e.g. F, t, r) with confidence intervals, effect sizes, degrees of freedom and P value noted<br>*Give P values as exact values whenever suitable.*
☒	☐	For Bayesian analysis, information on the choice of priors and Markov chain Monte Carlo settings
☒	☐	For hierarchical and complex designs, identification of the appropriate level for tests and full reporting of outcomes
☒	☐	Estimates of effect sizes (e.g. Cohen's d, Pearson's r), indicating how they were calculated

Our web collection on statistics for biologists contains articles on many of the points above.

Software and code

Policy information about availability of computer code

Data collection

Phylogenetic analysis and ASR
Sequences were collected from NCBI non-redundant protein sequences (nr) and UniprotKB. BLASTP searching tool was employed. Species tree was retrieved from TimeTree (http://www.timetree.org/).

Biochemical Assays
Cary-100 UV spectrophotometer, CaryWinUV Kinetics software
Oxygraph+ System (Hansatech Instruments Ltd), Oxytrace+ software
Stopped-flow SX20 Equipped with a PMT detector (Applied photophysics Surrey, UK)

NanoDSF
TycoTMNT.6 system (NanoTemper)

High-resolution Mass-spectrometry
X500B QTOF system (SCIEX, Framingham, MA 01701 USA) equipped with the Twin Sprayer ESI probe coupled to an ExionLCTM system (SCIEX). Proceesed using SCIEX OS software 2.1

Crystallography
Diffraction data were measured at the ESRF beamlines.

Data analysis	Phylogenetic analysis and ASR<br>MAFFT v7,RAXML v8.2.10, BOOSTER (online), Figtree v1.4.2, PAML 4.9a, Jalview, MEGA x<br><br>Analysis of Enzyme Kinetics and Activity Assays<br>GraphPad Prism 10<br><br>High-resolution Mass-spectrometry<br>SCIEX OS software 2.1<br><br>Crystallography<br>Diffraction data were processed with CCP4 Program Suite v8.0.017<br>Crystallographic refinement and model analysis were performed with CCP4 Program Suite v8.0.017, Phenix Software v1.21, COOT v0.9.8.92

For manuscripts utilizing custom algorithms or software that are central to the research but not yet described in published literature, software must be made available to editors and reviewers. We strongly encourage code deposition in a community repository (e.g. GitHub). See the Nature Portfolio guidelines for submitting code & software for further information.

Data

Policy information about availability of data

All manuscripts must include a data availability statement. This statement should provide the following information, where applicable:

- Accession codes, unique identifiers, or web links for publicly available datasets
- A description of any restrictions on data availability
- For clinical datasets or third party data, please ensure that the statement adheres to our policy

Source data are provided with this paper. Reconstructed protein sequences have been deposited in GenBank. The crystallographic data and atomic coordinates have been deposited in the Protein Data Bank. Accession codes are listed in the data availability statement. All constructs are available upon request.

Research involving human participants, their data, or biological material

Policy information about studies with human participants or human data. See also policy information about sex, gender (identity/presentation), and sexual orientation and race, ethnicity and racism.

Reporting on sex and gender	No human participant.
Reporting on race, ethnicity, or other socially relevant groupings	No human participant.
Population characteristics	No human participant.
Recruitment	No human participant.
Ethics oversight	No human participant.

Note that full information on the approval of the study protocol must also be provided in the manuscript.

Field-specific reporting

Please select the one below that is the best fit for your research. If you are not sure, read the appropriate sections before making your selection.

[x] Life sciences [ ] Behavioural & social sciences [ ] Ecological, evolutionary & environmental sciences

For a reference copy of the document with all sections, see nature.com/documents/nr-reporting-summary-flat.pdf

Life sciences study design

All studies must disclose on these points even when the disclosure is negative.

Sample size	No statistical methods were used to determine sample size. For all biochemical assays, we used a minimum sample size of n = 2 replicates and further confirmed reproducibility by replicating each experiment in two independent trials. This yields reproducible results based on our experience and is standard for enzymological assays.
Data exclusions	No data were systematically excluded.
Replication	All experimental findings were reproduced as the values stated in the figure legends. All replications were successful.
Randomization	No randomization was performed as this was not applicable to the experiments performed in this study. None of the experiments performed in this study involved allocating discrete samples or organisms to experimental groups.
Blinding	Investigators were not blinded for any of the experiments performed in this study. This was done for practical purposes, and is standard

practice for studies employing biochemistry,

Reporting for specific materials, systems and methods

We require information from authors about some types of materials, experimental systems and methods used in many studies. Here, indicate whether each material, system or method listed is relevant to your study. If you are not sure if a list item applies to your research, read the appropriate section before selecting a response.

Materials & experimental systems

n/a	Involved in the study
☒	☐ Antibodies
☒	☐ Eukaryotic cell lines
☒	☐ Palaeontology and archaeology
☒	☐ Animals and other organisms
☒	☐ Clinical data
☒	☐ Dual use research of concern
☒	☐ Plants

Methods

n/a	Involved in the study
☒	☐ ChIP-seq
☒	☐ Flow cytometry
☒	☐ MRI-based neuroimaging

Plants

Seed stocks	Not used
Novel plant genotypes	Not used
Authentication	Not used
